# Supplementary material for: Health-related quality of life in patients with implantable cardioverter defibrillators in Sweden: a cross-sectional observational trial
Source: BMJ Open. 2021 Jul 8;11(7):e047053. doi: 10.1136/bmjopen-2020-047053 (PMC8273444; doi:10.1136/bmjopen-2020-047053)
Supplement: Author's manuscript [file bmjopen-2020-047053.draft_revisions.pdf]

BMJ Open is committed to open peer review. As part of this commitment we make the peer review history of every article we publish publicly available.

When an article is published we post the peer reviewers' comments and the authors' responses online. We also post the versions of the paper that were used during peer review. These are the versions that the peer review comments apply to.

The versions of the paper that follow are the versions that were submitted during the peer review process. They are not the versions of record or the final published versions. They should not be cited or distributed as the published version of this manuscript.

BMJ Open is an open access journal and the full, final, typeset and author-corrected version of record of the manuscript is available on our site with no access controls, subscription charges or pay-per-view fees (http://bmjopen.bmj.com).

If you have any questions on BMJ Open's open peer review process please email info.bmjopen@bmj.com

BMJ Open

Health-related quality of life in patients with implantable cardioverter defibrillators – an observational trial

Journal:	*BMJ Open*
Manuscript ID	bmjopen-2020-047053
Article Type:	Original research
Date Submitted by the Author:	17-Nov-2020
Complete List of Authors:	Magnusson, Peter; Uppsala University,<br>Mattsson, Gustav; Uppsala University<br>Wallhagen, Marita; University of Gävle<br>Karlsson, Jan; Örebro Universitet
Keywords:	CARDIOLOGY, Adult cardiology < CARDIOLOGY, Heart failure < CARDIOLOGY, Pacing & electrophysiology < CARDIOLOGY

SCHOLARONE™
Manuscripts

I, the Submitting Author has the right to grant and does grant on behalf of all authors of the Work (as defined in the below author licence), an exclusive licence and/or a non-exclusive licence for contributions from authors who are: i) UK Crown employees; ii) where BMJ has agreed a CC-BY licence shall apply, and/or iii) in accordance with the terms applicable for US Federal Government officers or employees acting as part of their official duties; on a worldwide, perpetual, irrevocable, royalty-free basis to BMJ Publishing Group Ltd ("BMJ") its licensees and where the relevant Journal is co-owned by BMJ to the co-owners of the Journal, to publish the Work in this journal and any other BMJ products and to exploit all rights, as set out in our licence.

The Submitting Author accepts and understands that any supply made under these terms is made by BMJ to the Submitting Author unless you are acting as an employee on behalf of your employer or a postgraduate student of an affiliated institution which is paying any applicable article publishing charge ("APC") for Open Access articles. Where the Submitting Author wishes to make the Work available on an Open Access basis (and intends to pay the relevant APC), the terms of reuse of such Open Access shall be governed by a Creative Commons licence – details of these licences and which Creative Commons licence will apply to this Work are set out in our licence referred to above.

Other than as permitted in any relevant BMJ Author's Self Archiving Policies, I confirm this Work has not been accepted for publication elsewhere, is not being considered for publication elsewhere and does not duplicate material already published. I confirm all authors consent to publication of this Work and authorise the granting of this licence.

For peer review only

Health-related quality of life in patients with implantable cardioverter defibrillators – an observational trial

Peter Magnusson[1, 2] MD PhD; Gustav Mattsson[2] MD; Marita Wallhagen[3] PhD; Jan Karlsson[4] PhD

1. Cardiology Research Unit, Department of Medicine, Karolinska Institutet, Stockholm, SE-171 76, SWEDEN
2. Centre for Research and Development, Uppsala University/Region Gävleborg, Gävle, SE- 801 87, SWEDEN
3. Department of Building Engineering, Energy Systems and Sustainability Science, University of Gävle, Gävle, SE-80176, SWEDEN
4. University Health Care Research Center, Faculty of Medicine and Health, Örebro University, SE-70182 Örebro, SWEDEN

Corresponding author:

Peter Magnusson

Cardiology Research Unit/ Department of Medicine

Karolinska Institutet, Institution of Medicine, Solna

SE-171 76 Stockholm, SWEDEN

Phone: +46(0)705 089407 Fax: +46(0)26 154255

E-mail: peter.magnusson@regiongavleborg.se

Keywords: cardiomyopathy, implantable cardioverter defibrillator, quality of life, SF-36

Abbreviations: AF, atrial fibrillation; BP, bodily pain; CI, confidence interval; CRT, cardiac resynchronization therapy; ICD, implantable cardioverter defibrillator; GH, general health; HF, heart failure; HRQL, health related quality of life; MH, mental health; PF, physical functioning; RE, role emotional; RP, role physical; SCD, sudden cardiac death; SD, standard deviation; SF, social functioning; SF-36, short form 36; VT, vitality

Word count: 3357 (without title page, abstract, tables, figures, and references)

Short title: HRQL in ICD

For peer review only

Abstract

Objective Decisions regarding implantable cardioverter defibrillators (ICDs) must consider information about presumed health-related quality of life (HRQL). The purpose of the study was to assess HRQL in ICD patients and compare it to a Swedish age- and sex-matched population.

Methods and Results SF-36 questionnaires from ICD recipients implanted 2007-2017 (response rate 77.2%) were analyzed using Mann-Whitney U-test and effect size (ES). In total, 223 patients (mean age 71.1 ±9.7 years, 82.1% men) were included. In most SF-36 domains (physical functioning, role physical, general health, vitality, social functioning, and mental health), the score for ICD patients was significantly lower (ES range 0.23-0.41, i.e. small difference) than norms, except for bodily pain and role emotional. Both physical component summary (PCS) and the mental component summary (MCS) scores had ES=0.31. Men and women had similar scores. Primary and secondary prevention patients scored similarly, except for worse general health in primary prevention (p=0.016, ES=0.35). Atrial fibrillation was associated with worse physical functioning (ES=0.41) and PCS (ES=0.38). Appropriate therapy, inappropriate shock, or complications requiring surgery were not associated with lower scores in any domain. In primary prevention due to ischemic vs nonischemic cardiomyopathy no domain was significantly different. PCS decreased with higher age strata (p=0.002) in contrast to MCS (p=0.986).

Conclusion Patients with ICDs have lower physical and mental HRQL than age- and sex-matched norms; however, the effect sizes are small. HRQL is similar regardless of sex, primary/secondary prevention indication, appropriate therapy, inappropriate shock, or complications, but decreases with advancing age.

Strengths and limitations of this study

• Cross-sectional assessment of health-related quality of life in unselected ICD patients.

• Long-term ICD treatment, with associations of ICD outcomes and subgroup analyses.

• Generic health-related quality of life using SF-36 compared to age- and sex- matched population norms.

For peer review only

INTRODUCTION

An implantable cardioverter defibrillator (ICD) effectively terminates ventricular arrhythmias and protects from severe bradycardia. The ICD is a cornerstone in the treatment of heart failure with reduced ejection fraction but is also used for miscellaneous other conditions and is generally cost effective.[1–3] In addition to long-term protection from sudden cardiac death, ICDs can reduce symptoms of heart failure and prolong life when combined with cardiac resynchronization therapy (CRT).[2] In survivors of ventricular fibrillation or sustained ventricular arrhythmias with hemodynamic compromise, secondary prevention is an established indication, providing a reasonable life expectancy with good functional status for more than one year.[1,4] Using similar reasoning regarding life expectancy, primary prevention in high-risk patients is recommended.[1,4] Unfortunately, adverse events related to ICDs occur; the cumulative incidence at 12 years is about 20% inappropriate shock, 6% device-related infections, and 17% lead failure, and the incidence of complications seems to be underestimated in reports from registries.[5,6] The ICD affects several aspects of life, both professional life and leisure time activities. There may be restrictions related to driving, sports activities, and there is a risk for electromagnetic interference. ICD treatment has been associated with sleep disturbances, body-perception concerns, anxiety, and depression.

Patient-reported outcome measurements, such as health-related quality of life (HRQL), is recognized as a valuable research tool. It contributes information beyond echocardiographically assessed ejection fraction, biomarkers, or physician-assessed New York Heart Association (NYHA) functional class.[7,8] Several studies, mostly from controlled trials with interventions, have evaluated HRQL in ICD cohorts. The assessment of HRQL can be measured using a generic and/or a disease-specific questionnaire. The widely used short form (SF-36) often provides a basis for generic evaluation in diverse populations.[9,10] Whereas

For peer review only

worsening HRQL has been shown in numerous ICD cohorts, it remains largely unknown if this can be generalized into unselected ICD cohorts with long-term follow-up outside of a clinical trial.[11,12] Therefore, the primary aim of this study was to determine SF-36 scores in unselected ICD patients in comparison with age- and sex-matched general Swedish population norms. The secondary aims were to assess HRQL with regard to baseline atrial fibrillation (AF) or heart failure, appropriate ICD therapy, inappropriate ICD shock, device-related complications, and compare secondary versus primary indications.

METHODS

Design, setting, and selection

This retrospective observational study covered patients in Region Gävleborg, Sweden, who had an ICD implanted or underwent device replacement between 1st January 2007 and 1st January 2017. Eligible patients were identified through Provisio™, a software used for scheduling surgeries, with complete coverage of all device implants. Results from this cohort has been published previously and cover long-term follow-up with regard to appropriate therapy, complications, and mortality.[13]

Data were retrieved from electronic medical records (Melior™, Cerner Sverige AB, Stockholm) between March 2017 and February 2018 and evaluated according to a predefined protocol and imported from Excel™ 2010 (Microsoft Corporation, Redmond, WA) into SPSS™ version 22 (IBM, Armonk, NY) and Stata™ (StataCorp (2017), Stata Statistical Software: Release 15, College Station, TX) for statistical analyses.

Ethics and dissemination

For peer review only

The study complies with the Declaration of Helsinki and has been approved by the Ethical Review Board in Uppsala (document number 2018/416).

Statistical analyses

Data were described as frequencies, percentages, means including standard deviations (SD), and 95% confidence intervals (CIs) were used. The t-test was used for comparisons of continuous variables. Differences in SF-36 domains between groups were tested from the non-parametric Mann-Whitney U-test. The associations between age and HQRL were tested using Spearman's non-parametric correlation coefficient for analyses and Pearson´s correlation analysis to confirm results. Age was categorized into the four strata: 32-59 years, 60-69 years, 70-79 years, and ≥80 years and differences between age strata were analyzed using the Kruskal-Wallis non-parametric analysis of variance. The estimation of the magnitude of difference between group was further assessed by calculating effect sizes (ESs), that is, the mean difference, divided by the pooled standard deviation (Cohen's *d*). The interpretation of ES was described according to standard criteria: trivial (<0.20), small (0.20-0.49), moderate (0.50-0.79), and large (≥0.80).[14] All two-sided *p*-values <0.05 were considered statistically significant. The software programs Excel 2010 (Microsoft Corporation, Redmond, WA), SPSS version 22 (IBM, Armonk, NY), and SAS version 9.2 (SAS Institute Inc., Cary, NC) was used for analyses.

Definitions of variables

The baseline clinical characteristics of the patients were retrieved from medical records by physicians under supervision of an expert in the field (PM).

ICD therapy was defined as appropriate if an event of either antitachycardia pacing or capacitor discharge of 30-45 Joules due to an episode of ventricular arrhythmia above the

For peer review only

programmed detection interval and duration. ICD shock was classified as inappropriate if it was due to false detection or interpretation of myocardial signals (supraventricular tachycardia, T-wave oversensing) or signals outside the heart (caused by lead defect, myopotentials, electromagnetic interference).

Primary indication refers to patients who underwent ICD implant based on risk factors without any known *secondary* indication, i.e. survival of documented sustained ventricular arrhythmia with hemodynamic compromise including ventricular fibrillation.

AF or flutter had to be at least 30 seconds in duration and documented by 12-lead ECG. HF classification was based on systolic dysfunction with an ejection fraction ≤50%. The concept *complication* was restricted to events that required a surgical intervention related to the device system or the implant procedure, but excluded elective device replacement.

SF-36 domains and validation

The HRQL was assessed by the SF-36 health survey version 1.[15] This is a patient-reported multidimensional HRQL tool derived from the Medical Outcome Study in the 1990s.[10] It has been widely used for determination of the patient-perceived burden of different medical conditions and is considered a generic instrument. The instrument has 36 items, which measures eight domains that cover both physical and mental health domains: physical functioning (PF), role-physical (RP), bodily pain (BP), general health (GH), vitality (VT), social functioning (SF), role-emotional (RE), and mental health (MH). In each domain, the scores range from 0 to 100 and higher values indicate better HRQL. Based on these domains, the physical component summary (PCS) and the mental component summary (MCS) can be calculated using norm-based scoring with a mean of 50. A score above 50 would be interpreted as better HRQL than the general Swedish population. The validation of the

For peer review only

Swedish SF-36 yielded Cronbach's α between 0.79 (role-emotional) and 0.93 (bodily pain) for the domains.[10]

The SF-36 profile in the study group was compared to a general population sample which was randomly selected from the Swedish SF-36 normative database (n=8,930; response rate 68%).[15] The normative sample (validated in Sweden 1991-92) was matched on sex and age and comprised 171 persons (141 males) with a mean age of 68.8 years (SD 11.1).

Patient and public involvement

Patients were not involved in the design, or conduct, or reporting, or dissemination plan of our research.

Results

Cohort characteristics and follow-up

The SF-36 questionnaire was returned by 223 ICD patients which corresponds to a response rate of 77.2%. The mean age was similar between those who responded and those who did not (p=0.276). The same was seen between sexes (p=0.255). Figure 1 depicts the flow-chart of the emerged cohort. The majority was men 82.1% (n=183) and 60.1% (n=134) had an ICD due to primary prevention. All leads in the device systems were implanted transvenously. The baseline characteristics of the patients are summarized in Table 1.

A total of 52 patients (23.3%) experienced appropriate ICD therapy, whereas 14 (6.3%) experienced inappropriate ICD shock during the follow-up period. Complications which required surgical intervention occurred in 39 patients (17.5%). Among patients with cardiomyopathy, 76 had ischemic disease and 56 had no known underlying ischemic disease.

For peer review only

The ages ranged from 32 to 90 years with a median age of 71.2 years. The mean age was 71.1 SD 9.7 years. Men and women had similar mean ages (71.1 vs 70.9 years). Similarly, the mean ages were not different for primary vs secondary prevention (70.3 vs 72.3 years; p=0.138), a history of appropriate ICD therapy (70.7 vs 72.3 years; p=0.286), inappropriate shock (71.1 vs 71.0 years; p=0.967), or a complication requiring surgery (71.1 vs 70.9 years; p=0.907). On the contrary, patients with ischemic cardiomyopathy were older than nonischemic cardiomyopathy patients (72.9 vs 66.9 years; p<0.001) as were patients with a history of AF at baseline (73.4 vs 70.1 years; p=0.024).

HRQL in the ICD cohort compared to general population norms

In six out of eight SF-36 domains, there was a significantly lower score in ICD patients compared to sex- and aged-matched population norms (Table 2 and Figure 2).

There was a statistically significant (p<0.05) difference for PF, RP, GH, VT, SF, and MH, but not for BP and RE. The effect size among the significantly lower domains ranged from 0.23 (RP) to 0.41 (GH). Both component scores were significantly lower and the effect sizes similar, PCS (0.31) and MCS (0.31). Thus, all effect sizes were classified as small. Men and women reported similar SF-36 scores with nonsignificant differences in all domains including the component scores; PCS 41.0 SD 12.4 vs 39.5 SD 12.0 (p=0.406) and MCS 48.7 SD 10.9 vs 48.3 SD 10.5 (p=0.644)

Increasing age was associated with significantly lower scores on PF (r=-0.36; p<0.001), RP (r=-0.25; p<0.001), BP (r=-0.20; p=0.003), RE (r=-0.16; p=0.017), and PCS (r=-0.28; p<0.001).

SF-36 scores for the following age strata: 32-59, 60-69, 70-79, and ≥80 years, are reported in Table 3. Kruskal-Wallis test showed significantly lower scores for PF, RP, and PCS, while BP and RE showed a trend toward lower scores.

For peer review only

HRQL subgroup analyses

The subgroup analyses for AF, complications, appropriate therapy, and inappropriate shocks are reported in Table 4. Patients with primary-prevention ICDs were compared to secondary prevention indications. The age was similar, 70.3 vs 72.3 years. Primary-prevention patients scored lower GH (p=0.016; ES 0.35) and there was a trend toward lower VT (p=0.097; ES=0.21), but otherwise no significant differences.

A history of AF at baseline was associated with significantly lower PF (p=0.004; ES=0.41) and a trend toward lower BP (p=0.083; ES=0.27) and VT (p=0.057; ES=0.31). This translated into lower PCS (p=0.013; ES 0.38) but similar MCS (p=0.928). However, the mean age of patients with AF was slightly higher (73.4 vs 70.1 years; p=0.024).

The mean age of patients who experienced appropriate ICD therapy patients was similar to patients who did not receive appropriate shocks (72.3 vs 70.7 years) and all SF-36 domains were similar without any significant differences or trends. The group who experienced inappropriate ICD therapy was small, but had a similar mean age and no significant differences or trends with regard to any SF-36 domain. Furthermore, the group who required surgical intervention due to a complication had a similar mean age and SF-36 scores without significant differences.

In the primary-prevention group, nonischemic vs ischemic cardiomyopathy were compared. Notably, patients with an ischemic etiology were older (72.9 vs 66.9 years) but no SF-36 domain showed significantly different scores (RP showed a trend of lower score in ischemic cardiomyopathy, p=0.067; ES=0.31).

DISCUSSION

For peer review only

This Swedish cohort of general ICD patients report worse HRQL than age- and sex-matched population norms. Except for BP and RE, all domains were significantly lower although effect sizes were considered small. The component score, PCS and MCS, showed an equally small effect size.

Similar component score

In a recently published Danish study based on unselected ICD recipients, PCS (41.1 SD 10.3) and MCS (45.7 SD 10.8) at a mean follow-up of 7 years, were similar to our findings; poor PF, RP, GH, and VT were associated with higher long-term mortality.[16] Early randomized controlled trials with shorter follow-up (1-3 years) reported similar findings.[17,18] In the MADIT II , PCS was associated with an 89% higher mortality, while MCS showed 39% higher mortality.[19] US data from the INTRINSIC RV trial found improvement of SF-score over the first year and at 12 months, PCS was 42.2 and MCS 51.1, which is also similar to our results.[20]

Heart failure and HRQL

Systolic heart failure was the ICD indication for the vast majority of patients in our cohort, a third of whom received a CRT-D device. Several studies have determined that heart failure is a main determinant of poor HRQL.[21]

In a systematic review reviewing many studies based on SF-36 poor physical health status predicted adverse coronary artery disease and HF prognosis.[21] Using both disease-specific and generic test instruments, HF studies clearly revealed poor HRQL, but , poor mental status had little impact. Moreover, follow-up time did not influence the predictive value of health status. In another review, those who reported poor disease-specific health status, in particular HF patients, had increased mortality (hazard ratio 1.39) compared with those with moderate/good health status.[11] Systematic reviews in this field are hampered by severe

For peer review only

heterogeneity across populations, different follow-up times, and diverse outcome measurements. Still, HF has repeatedly been shown to be a major determinant of poor health status.[12,22] Underlying cardiac disease and comorbidities influence HRQL and mortality.[12,23] A Cochrane review of nonischemic HF concluded: “using ICD therapy probably has little or no effect on quality of life.”[24] This view is further strengthened by the fact that various ICD patients without structural heart disease report similar SF-36 scores as the general populations.[25,26]

Atrial fibrillation

A systematic review of several studies demonstrated poorer HRQL in AF patients than healthy controls or general populations.[27] Notably, studies on AF and HRQL are diverse and often from specific cohorts as part of an evaluation of an intervention. Such studies are prone to selection bias because they likely reflect highly symptomatic patients. Nevertheless, more general AF patients likewise show lower HRQL.[28,29] With a qualitative exploratory approach, the various symptoms have been further elucidated.[30] AF patients consist of subgroups including those with paroxysmal, persistent, and permanent AF; and even within groups, the burden of symptoms may vary substantially. AF is common in patients with heart failure and thus prevalent in ICD cohorts. In our study, the comparison among those with/without a history of AF at baseline showed that the association of AF with poor HRQL was more pronounced for the physical domains, but this may be at least partly explained by older age.

Age

As expected, age correlated with lower HQRL scores on physical domains. The four age strata showed that PCS deteriorated with older age and demonstrated significantly lower

For peer review only

scores in the analyses. This is likely due to the burden of heart failure, AF, and other comorbidities. Interestingly, mental health domains were not affected and remained consistent throughout the age strata. It can be speculated that patients selected for ICD therapy reflect a group with relatively good mental health status.

Primary versus secondary indication

The proportion of primary prophylactic ICDs was low. This reflect a historical view on indication and possibly a more conservative approach towards primary prevention. Overall, the HRQL was similar and solely the GH domain was significantly lower in the primary prevention group. The coping strategy may differ between primary and secondary prevention patients. A survivor of a life-threatening arrhythmia may feel gratitude but be prone to anxiety and depression. A lower SF-36 score (all domains except BP) in primary vs secondary prevention has been shown, which underlines the vulnerability in the primary-prevention group.[31] The differences may also be attributed to baseline characteristics; HF is a decisive factor for ICD implantation and translates into worse HRQL scores. Secondary- vs primary- prevention patients are likely to have different expectations and acceptance of ICD therapy and its related complications.

ICD shocks and complications

In the three separate subgroup analyses with regard to appropriate ICD therapy, inappropriate ICD shock, and complications requiring surgery, no significant differences in the SF-36 domains were observed. This is somewhat reassuring, at least on a group level. Data from the INTRINSIC RV trial found no significant difference in HRQL between patients with and without ICD shocks.[20]

For peer review only

In a Danish cohort, HF was associated with poor scores on all SF-36 domains, while age and ICD shocks correlated less. Notably, on depression/anxiety-specific instruments, ICD shocks were a determinant for outcome. This suggests that it is the comorbidity burden, rather than the ICD itself, that determines the patients' HRQL and despite the fact that device-related complications can be distressing to individuals, ICDs are well tolerated overall. Of course, multiple ICD shocks may translate into worse HRQL, especially anxiety.[32] Our study was unable to detect a difference between patients with ICD shocks and those without. Several explanations have been suggested for this: lower incidence of shocks, ability to cope with complications over time, generic instruments not specifically designed for ICDs, and the overall security and reassurance that comes from an ICD. Thus, it should be highlighted that careful device programming and management to avoid shocks and complications are crucial for optimal care.

Gender perspectives

The vast majority (82%) of patients in our study were men. This is in line with a review of 19 studies, in which the percentage of men ranged from 54% to 83%. We found similar SF-scores in men and women. In another study, only PF and VT were lower among women, although the difference was small, while for women, NYHA-class and Type D personality were strong determinants of poor HRQL.[23] Women may be more prone to anxiety and to score lower also on some SF-36 domains (PF, SF, MH) regardless of a history of ICD shock.[33] There needs to be an increased awareness of female candidates for ICD therapy, and our study support the fact that there are no overall sex differences with regard to generic HRQL.

Clinical perspective

For peer review only

HRQL assessment has the potential to provide insights helpful to the overall management of ICD patients, including selection of ICD candidates, and the reduction of hospitalization and mortality.[34] Patients with ICDs have poorer general HRQL than the general population and increasing age and HF are strong determinants of worse HRQL. The underlying burden of comorbidities is the main driver for low HRQL rather than the ICD itself. Concerning quality of life improvement, overall management of the cardiac disease manifestations and comorbidities is important but challenging despite advances in many therapeutic fields. In fact, according to a meta-analysis there have been slight improvements regarding physical components, but not for mental components.[35] However, a comprehensive cardiac rehabilitation program in ICD patients improved both GH and MH.[34] Even though ICD shocks and complications can be devastating for an individual, the overall perception is that an ICD is generally well tolerated and does not affect generic HRQL. Individual assessment of benefits and risks, not gender category, should be part of the risk assessment strategy.

Furthermore, complications of ICD therapy do not have a significant impact on HRQL and should not be a reason to refrain from offering an ICD to otherwise eligible patients. Nevertheless, ICD is lifelong therapy and clinicians should regularly monitor the patient's ICD as well as quality of life as part of follow-up.

Limitations

The major strength of our study is the use of a "real-world" cohort, rather than strict inclusion/exclusion criteria used in randomized controlled trials, although this implies more heterogeneity than randomized controlled studies. The ICD patients included in our study come from a cohort of unselected patients without referral center bias. The SF-36 scores in the study group were compared with age- and sex-matched general population norms, that

For peer review only

although more than 20 years old, likely still represent current status. The observational design reflects common practice outside a clinical trial, but may hampered by lack of details in assessment of clinical profiles even though medical records were scrutinized. The proportion who responded to the survey was comparatively high, which implies less risk of differential bias.

The HRQL assessment comes from a cross-sectional cohort studied long after initial implant and likely reflects views from a more stabilized phase of life as an ICD patient. Nevertheless, the long-term impact on HRQL reported over the life of ICD patients remains unknown. We lack information about personality traits and socioeconomic factors, which may affect self-reported health. SF-36 is a well-validated questionnaire that allows for comparisons on a group level but is not specifically developed for disease-specific assessment. The study design has limitations with respect to determining causal pathways and merely show associations. Therefore, it should be interpreted with caution.

Conclusions

ICD patients have lower physical and mental HRQL, assessed by SF-36, than age- and sex-matched norms, although effect sizes are small. HRQL is similar regardless of sex, primary- or secondary-prevention indication, appropriate therapy, inappropriate shock, or complications but decreases with older age.

DECLARATIONS

Ethics: The study complies with the Declaration of Helsinki and has been approved by the Ethical Review Board in Uppsala (document number 2018/416).

For peer review only

Consent for publication: Not applicable.

Acknowledgements: The authors acknowledge editing by Jo Ann LeQuang of LeQ Medical who reviewed the manuscript for American English use. Thanks to Kristina Bergström for her help with data collection through the questionnaires. Region Gävleborg funded this research project.

Author contributions: Peter Magnusson: design, collection and interpretation of data, statistical analyses, writing the article. Gustav Mattsson: collection and interpretation of data, statistical analyses, critical revision writing the article. Marita Wallhagen: interpretation of data, critical revision. Jan Karlsson: statistical analyses, interpretation of data, critical revision. All authors approved the manuscript for submission.

Funding: This study had no external funding.

Competing interests: Peter Magnusson has received speaker fees or grants from Abbott, Alnylam, Amicus Therapeutics, Bayer, AstraZeneca, BMS, Boehringer-Ingelheim, Internetmedicin, Lilly, MSD, Novo Nordisk, Octopus Medical, Pfizer, Vifor Pharma, and Zoll.

Gustav Mattsson has received speaker fee from Alnylam, Internetmedicin, and MSD.

Marita Wallhagen and Jan Karlsson disclose no conflict of interest.

No external financial support or funding was received for this study.

Patient consent for publication: Not applicable.

Ethics approval: Approved by the Ethical Review Board in Uppsala (document number 2018/416)

Data sharing statement: No additional data sharing available.

For peer review only

Patient and public involvement: Patients were not involved in the design, or conduct, or reporting, or dissemination plan of our research.

ORCID: Peter Magnusson: https://orcid.org/0000-0001-7906-7782

Gustav Mattsson: https://orcid.org/0000-0002-4317-0443

Marita Wallhagen: https://orcid.org/0000-0001-8413-3975

For peer review only

References

1. Priori SG, Blomström-Lundqvist C, Mazzanti A, et al. 2015 ESC Guidelines for the management of patients with ventricular arrhythmias and the prevention of sudden cardiac death: The Task Force for the Management of Patients with Ventricular Arrhythmias and the Prevention of Sudden Cardiac Death of the European Society of Cardiology (ESC). Endorsed by: Association for European Paediatric and Congenital Cardiology (AEPC). Eur Heart J 2015;36(41):2793–867.

2. Goldenberg I, Gillespie J, Moss AJ, et al. Long-term benefit of primary prevention with an implantable cardioverter-defibrillator: an extended 8-year follow-up study of the Multicenter Automatic Defibrillator Implantation Trial II. Circulation 2010;122(13):1265–71.

3. Smith T, Jordaens L, Theuns DAMJ, van Dessel PF, Wilde AA, Hunink MGM. The cost-effectiveness of primary prophylactic implantable defibrillator therapy in patients with ischaemic or non-ischaemic heart disease: a European analysis. Eur Heart J 2013;34(3):211–9.

4. Al-Khatib SM, Stevenson WG, Ackerman MJ, et al. 2017 AHA/ACC/HRS Guideline for Management of Patients With Ventricular Arrhythmias and the Prevention of Sudden Cardiac Death: A Report of the American College of Cardiology/American Heart Association Task Force on Clinical Practice Guidelines and the Heart Rhythm Society. J Am Coll Cardiol 2018;72(14):e91–220.

5. van der Heijden AC, Borleffs CJW, Buiten MS, et al. The clinical course of patients with implantable cardioverter-defibrillators: Extended experience on clinical outcome, device replacements, and device-related complications. Heart Rhythm 2015;12(6):1169–76.

6. Kirkfeldt RE, Johansen JB, Nohr EA, Jørgensen OD, Nielsen JC. Complications after cardiac implantable electronic device implantations: an analysis of a complete, nationwide cohort in Denmark. Eur Heart J 2014;35(18):1186–94.

7. Rector TS, Anand IS, Cohn JN. Relationships between clinical assessments and patients' perceptions of the effects of heart failure on their quality of life. J Card Fail 2006;12(2):87–92.

8. Luther SA, McCullough PA, Havranek EP, et al. The relationship between B-type natriuretic peptide and health status in patients with heart failure. J Card Fail 2005;11(6):414–21.

9. McHorney CA, Ware JE, Raczek AE. The MOS 36-Item Short-Form Health Survey (SF-36): II. Psychometric and clinical tests of validity in measuring physical and mental health constructs. Med Care 1993;31(3):247–63.

10. McHorney CA, Ware JE, Lu JF, Sherbourne CD. The MOS 36-item Short-Form Health Survey (SF-36): III. Tests of data quality, scaling assumptions, and reliability across diverse patient groups. Med Care 1994;32(1):40–66.

11. Mastenbroek MH, Versteeg H, Zijlstra W, Meine M, Spertus JA, Pedersen SS. Disease-specific health status as a predictor of mortality in patients with heart failure: a systematic literature review and meta-analysis of prospective cohort studies. Eur J Heart Fail 2014;16(4):384–93.

12. Hoogwegt MT, Kupper N, Jordaens L, Pedersen SS, Theuns DAMJ. Comorbidity burden is associated with poor psychological well-being and physical health status in patients with an implantable cardioverter-defibrillator. Europace 2013;15(10):1468–74.

13. Mattsson G, Magnusson P. Long-term follow-up of implantable cardioverter defibrillator patients with regard to appropriate therapy, complications, and mortality. Pacing Clin Electrophysiol 2020;43(2):245–53.

14. Cohen J. Statistical power analysis for the behavioral sciences. 2nd ed. Hillsdale, N.J: L. Erlbaum Associates; 1988.

15. Sullivan M, Karlsson J, Ware JE. The Swedish SF-36 Health Survey--I. Evaluation of data quality, scaling assumptions, reliability and construct validity across general populations in Sweden. Soc Sci Med 1995;41(10):1349–58.

16. van Veen B, Andersen CM, Johansen JB, Theuns DA, Pedersen SS. Patient-Reported Quality of Life as a Predictor of Mortality and Ventricular Tachyarrhythmia's During 7 Years' Follow-Up in Patients With an Implantable Cardioverter Defibrillator (from the MIDAS Study). Am J Cardiol 2019;123(4):605–10.

17. Steinberg JS, Joshi S, Schron EB, et al. Psychosocial status predicts mortality in patients with life-threatening ventricular arrhythmias. Heart Rhythm 2008;5(3):361–5.

18. Kao C-W, Friedmann E, Thomas SA. Quality of life predicts one-year survival in patients with implantable cardioverter defibrillators. Qual Life Res 2010;19(3):307–15.

For peer review only

19. Piotrowicz K, Noyes K, Lyness JM, et al. Physical functioning and mental well-being in association with health outcome in patients enrolled in the Multicenter Automatic Defibrillator Implantation Trial II. Eur Heart J 2007;28(5):601–7.

20. Gopinathannair R, Lerew DR, Cross NJ, Sears SF, Brown S, Olshansky B. Longitudinal changes in quality of life following ICD implant and the impact of age, gender, and ICD shocks: observations from the INTRINSIC RV trial. J Interv Card Electrophysiol 2017;48(3):291–8.

21. Mommersteeg PMC, Denollet J, Spertus JA, Pedersen SS. Health status as a risk factor in cardiovascular disease: a systematic review of current evidence. Am Heart J 2009;157(2):208–18.

22. Johansen JB, Pedersen SS, Spindler H, Andersen K, Nielsen JC, Mortensen PT. Symptomatic heart failure is the most important clinical correlate of impaired quality of life, anxiety, and depression in implantable cardioverter-defibrillator patients: a single-centre, cross-sectional study in 610 patients. Europace 2008;10(5):545–51.

23. Habibović M, van den Broek KC, Theuns DAMJ, et al. Gender disparities in anxiety and quality of life in patients with an implantable cardioverter-defibrillator. Europace 2011;13(12):1723–30.

24. El Moheb M, Nicolas J, Khamis AM, Iskandarani G, Akl EA, Refaat M. Implantable cardiac defibrillators for people with non-ischaemic cardiomyopathy. Cochrane Database Syst Rev 2018;12:CD012738.

25. Probst V, Plassard-Kerdoncuf D, Mansourati J, et al. The psychological impact of implantable cardioverter defibrillator implantation on Brugada syndrome patients. Europace 2011;13(7):1034–9.

26. Christiaans I, van Langen IM, Birnie E, Bonsel GJ, Wilde AAM, Smets EMA. Quality of life and psychological distress in hypertrophic cardiomyopathy mutation carriers: a cross-sectional cohort study. Am J Med Genet A 2009;149A(4):602–12.

27. Thrall G, Lane D, Carroll D, Lip GYH. Quality of life in patients with atrial fibrillation: a systematic review. Am J Med 2006;119(5):448.e1-19.

28. Dorian P, Jung W, Newman D, et al. The impairment of health-related quality of life in patients with intermittent atrial fibrillation: implications for the assessment of investigational therapy. J Am Coll Cardiol 2000;36(4):1303–9.

29. van den Berg MP, Hassink RJ, Tuinenburg AE, et al. Quality of life in patients with paroxysmal atrial fibrillation and its predictors: importance of the autonomic nervous system. Eur Heart J 2001;22(3):247–53.

30. Stridsman M, Strömberg A, Hendriks J, Walfridsson U. Patients' Experiences of Living with Atrial Fibrillation: A Mixed Methods Study. Cardiol Res Pract 2019;2019:6590358.

For peer review only

31. Kikkenborg Berg S, Caspar Thygesen L, Hastrup Svendsen J, Vinggaard Christensen A, Zwisler A-D. Anxiety predicts mortality in ICD patients: results from the cross-sectional national CopenHeartICD survey with register follow-up. Pacing Clin Electrophysiol 2014;37(12):1641–50.

32. Redhead AP, Turkington D, Rao S, Tynan MM, Bourke JP. Psychopathology in postinfarction patients implanted with cardioverter-defibrillators for secondary prevention. A cross-sectional, case-controlled study. J Psychosom Res 2010;69(6):555–63.

33. Spindler H, Johansen JB, Andersen K, Mortensen P, Pedersen SS. Gender differences in anxiety and concerns about the cardioverter defibrillator. Pacing Clin Electrophysiol 2009;32(5):614–21.

34. Kosiborod M, Soto GE, Jones PG, et al. Identifying heart failure patients at high risk for near-term cardiovascular events with serial health status assessments. Circulation 2007;115(15):1975–81.

35. Kao C-W, Chen M-Y, Chen T-Y, Lin P-H. Effect of psycho-educational interventions on quality of life in patients with implantable cardioverter defibrillators: a meta-analysis of randomized controlled trials. Health Qual Life Outcomes 2016;14(1):138.

For peer review only

● **Table 1.** Characteristics at ICD implant of 223 patients who completed the SF-36.

Patients	223
Mean age at implant	64.1 ± 10.9 years
Mean age at SF-36	71.1 ± 9.7 years
Females	40 (17.9%)
Device type	
ICD-VR	36 (16.1%)
ICD-DR	112 (50.2%)
CRT-D	75 (33.6%)
Hypertension	104 (46.6%)
Diabetes mellitus	47 (21.1%)
Renal failure*	27 (12.1%)
Atrial fibrillation	62 (27.8%)
Beta-blockers	201 (90.1%)
ACE-i/ARB	193 (86.5%)
MRA	104 (46.6%)

Data presented as frequencies (percentage in parenthesis).

ACE-I, angiotensin converting enzyme inhibitor; ARB, angiotensin receptor blockers; CRT-D, cardiac resynchronization therapy defibrillator; ICD-DR, dual lead implantable cardioverter defibrillator; ICD-VR, single lead implantable cardioverter defibrillator; MRA, mineralcorticoid receptor antagonist

*defined as S-Creatinine ≥130 µmol/L

For peer review only

• **Table 2:** SF-36 score in patients with ICDs compared to general Swedish population norms.

**SF-36 domains**	**Cohort mean**	**SD**	**95% CI**	**Effect size**	**Norm mean**	**SD**	**95% CI**	***p*-value**
Physical functioning	65.1	28.1	61.4-68.8	0.38	75.5	26.0	71.5-79.6	**0.0002**
Role physical	56.9	44.1	50.9-62.8	0.23	66.7	40.4	60.2-73.2	**0.0466**
Bodily pain	71.9	29.9	67.9-75.8		71.0	27.2	66.9-75.1	0.6395
General health	56.2	21.7	53.3-59.1	0.41	65.9	25.3	61.9-70.0	**<0.0001**
Vitality	58.0	24.8	54.7-61.3	0.39	68.1	27.2	63.7-72.4	**<0.0001**
Social functioning	80.1	24.4	76.8-83.3	0.25	85.9	22.9	82.4-89.4	**0.0025**
Role emotional	72.2	39.9	66.9-77.6		76.7	35.7	71.1-82.4	0.3730
Mental health	76.2	19.4	73.6-78.8	0.38	83.8	20.9	80.5-87.2	**<0.0001**
Physical Component Summary	40.7	12.3	39.0-42.4	0.31	44.4	11.6	42.4-46.4	**0.0052**
Mental Component Summary	48.6	10.8	47.2-50.1	0.31	51.9	10.5	50.1-53.8	**0.0010**

For peer review only

● **Table 3:** SF-36 score in patients with implantable cardioverter defibrillators stratified by age.

	**32-59** (n=25)	**60-69** (n=72)	**70-79** (n=76)	**80+** (n=50)	
**SF-36 domains**	Mean (SD)	Mean (SD)	Mean (SD)	Mean (SD)	*p*-value*
**PF**	79.4 (25.4)	71.9 (26.3)	62.7 (29.0)	51.4 (24.4)	**<0.0001**
**RP**	80.0 (36.8)	60.0 (44.9)	56.0 (43.9)	40.9 (41.6)	**0.0050**
**BP**	80.8 (29.3)	74.4 (30.9)	71.5 (28.8)	64.2 (29.2)	0.0780
**GH**	57.5 (21.2)	56.0 (22.6)	56.2 (21.9)	55.7 (20.7)	0.9827
**VT**	64.6 (20.4)	59.7 (27.5)	56.5 (23.8)	54.3 (23.9)	0.2363
**SF**	84.0 (22.7)	79.3 (27.9)	80.3 (22.1)	78.8 (23.5)	0.8341
**RE**	82.7 (33.5)	75.6 (38.6)	73.6 (39.1)	59.7 (44.0)	0.0773
**MH**	77.8 (18.2)	77.2 (21.2)	74.4 (18.8)	76.8 (18.3)	0.6190
**PCS**	46.6 (11.1)	42.2 (12.7)	40.0 (11.9)	36.0 (11.4)	**0.0021**
**MCS**	48.6 (10.3)	48.3 (12.0)	48.6 (10.4)	49.3 (10.1)	0.9859

For peer review only

● **Table 4:** Subgroup analyses of ICD patients.

ES=effect size *Effect sizes were *lower* when atrial fibrillation was present.

SF-36 domains	Atrial fibrillation*		Complications		Appropriate therapy		Inappropriate shock	
	p-value	ES	*p*-value	ES	*p*-value	ES	*p*-value	ES
Physical functioning	**0.004***	0.41	0.500		0.947		0.592	
Role physical	0.128		0.210		0.897		0.428	
Bodily pain	**0.083***	0.27	0.167		0.544		0.381	
General health	0.606		0.190		0.863		0.921	
Vitality	**0.057***	0.31	0.321		0.919		0.765	
Social functioning	0.669		0.910		0.943		0.944	
Role emotional	0.313		0.972		0.796		0.955	
Mental health	0.912		0.769		0.747		0.972	
Physical Component Summary	**0.013***	0.38	0.107		0.696		0.464	
Mental Component Summary	0.923		0.902		0.772		0.375	

For peer review only

Caption:

Figure 1. Flowchart.

Figure 2. Bar chart: Health-related quality of life among ICD patients compared to age- and sex-matched norms.

For peer review only

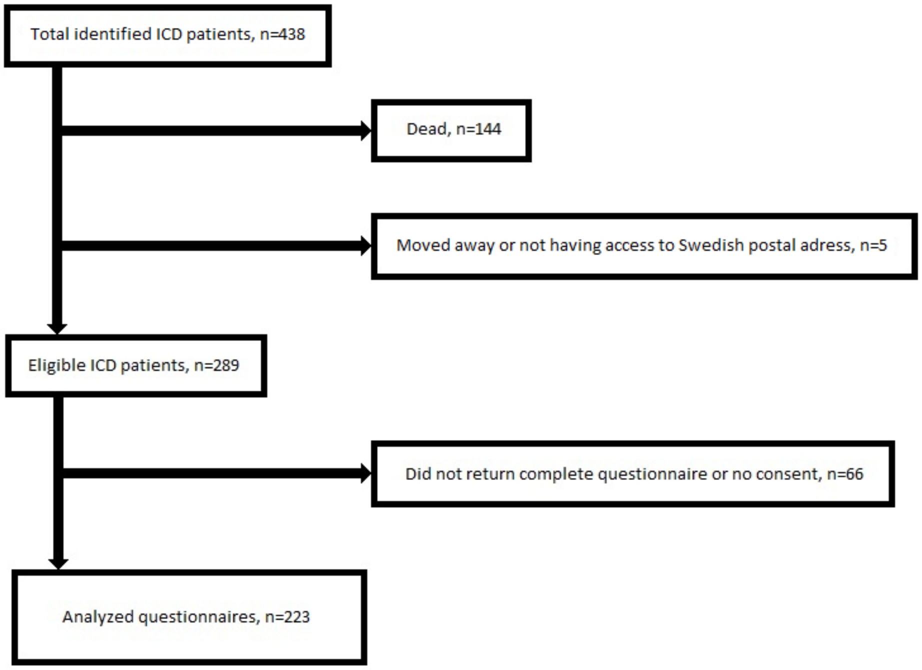

66x49mm (300 x 300 DPI)

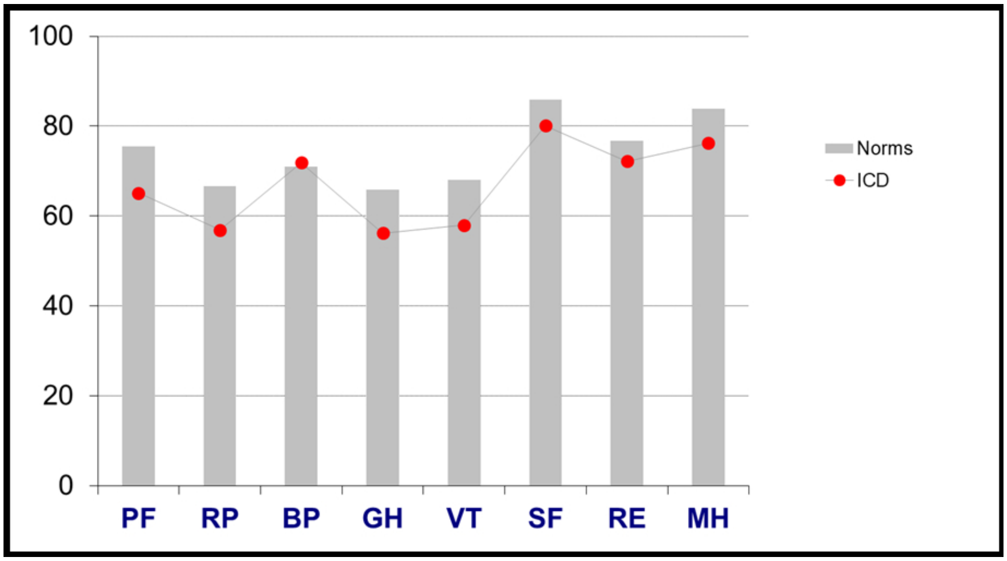
100
80
60
40
20
0
PF
RP
BP
GH
VT
SF
RE
MH
Norms
ICD

BMJ Open

Health-related quality of life in patients with implantable cardioverter defibrillators in Sweden – a cross-sectional observational trial

Journal:	*BMJ Open*
Manuscript ID	bmjopen-2020-047053.R1
Article Type:	Original research
Date Submitted by the Author:	22-Feb-2021
Complete List of Authors:	Magnusson, Peter; Uppsala University,<br>Mattsson, Gustav; Uppsala University<br>Wallhagen, Marita; University of Gävle<br>Karlsson, Jan; Örebro Universitet
**Primary Subject Heading**:	Cardiovascular medicine
Secondary Subject Heading:	Cardiovascular medicine
Keywords:	CARDIOLOGY, Adult cardiology < CARDIOLOGY, Heart failure < CARDIOLOGY, Pacing & electrophysiology < CARDIOLOGY

SCHOLARONE™
Manuscripts

I, the Submitting Author has the right to grant and does grant on behalf of all authors of the Work (as defined in the below author licence), an exclusive licence and/or a non-exclusive licence for contributions from authors who are: i) UK Crown employees; ii) where BMJ has agreed a CC-BY licence shall apply, and/or iii) in accordance with the terms applicable for US Federal Government officers or employees acting as part of their official duties; on a worldwide, perpetual, irrevocable, royalty-free basis to BMJ Publishing Group Ltd ("BMJ") its licensees and where the relevant Journal is co-owned by BMJ to the co-owners of the Journal, to publish the Work in this journal and any other BMJ products and to exploit all rights, as set out in our licence.

The Submitting Author accepts and understands that any supply made under these terms is made by BMJ to the Submitting Author unless you are acting as an employee on behalf of your employer or a postgraduate student of an affiliated institution which is paying any applicable article publishing charge ("APC") for Open Access articles. Where the Submitting Author wishes to make the Work available on an Open Access basis (and intends to pay the relevant APC), the terms of reuse of such Open Access shall be governed by a Creative Commons licence – details of these licences and which Creative Commons licence will apply to this Work are set out in our licence referred to above.

Other than as permitted in any relevant BMJ Author's Self Archiving Policies, I confirm this Work has not been accepted for publication elsewhere, is not being considered for publication elsewhere and does not duplicate material already published. I confirm all authors consent to publication of this Work and authorise the granting of this licence.

For peer review only

Health-related quality of life in patients with implantable cardioverter defibrillators in Sweden – a cross-sectional observational trial

Peter Magnusson[1, 2] MD PhD; Gustav Mattsson[2] MD; Marita Wallhagen[3] PhD; Jan Karlsson[4] PhD

1. Cardiology Research Unit, Department of Medicine, Karolinska Institutet, Stockholm, SE-171 76, SWEDEN
2. Centre for Research and Development, Uppsala University/Region Gävleborg, Gävle, SE- 801 87, SWEDEN

3. Department of Building Engineering, Energy Systems and Sustainability Science, University of Gävle, Gävle, SE-80176, SWEDEN

4. University Health Care Research Center, Faculty of Medicine and Health, Örebro University, SE-70182 Örebro, SWEDEN

Corresponding author:

Peter Magnusson

Cardiology Research Unit/ Department of Medicine

Karolinska Institutet, Institution of Medicine, Solna

SE-171 76 Stockholm, SWEDEN

Phone: +46(0)705 089407 Fax: +46(0)26 154255

E-mail: peter.magnusson@regiongavleborg.se

Keywords: cardiomyopathy, implantable cardioverter defibrillator, quality of life

Abbreviations: AF, atrial fibrillation; BP, bodily pain; CI, confidence interval; CRT, cardiac resynchronization therapy; ICD, implantable cardioverter defibrillator; GH, general health; HF, heart failure; HRQL, health related quality of life; MH, mental health; PF, physical functioning; RE, role emotional; RP, role physical; SCD, sudden cardiac death; SD, standard deviation; SF, social functioning; SF-36, short form 36; VT, vitality

Word count: 3357 (without title page, abstract, tables, figures, and references)

Short title: HRQL in ICD

For peer review only

Abstract

Objective Decisions regarding implantable cardioverter defibrillators (ICDs) must consider information about presumed health-related quality of life (HRQL). The purpose of the study was to assess HRQL in ICD patients and compare it to a Swedish age- and sex-matched population.

Methods SF-36 questionnaires from ICD recipients implanted 2007-2017 (response rate 77.2%) were analyzed using Mann-Whitney U-test and effect size (ES).

Results In total, 223 patients (mean age 71.1 ±9.7 years, 82.1% men) were included. In most SF-36 domains (physical functioning, role physical, general health, vitality, social functioning, and mental health), the score for ICD patients was significantly lower (ES range 0.23-0.41, i.e. small difference) than norms, except for bodily pain and role emotional. Both physical component summary (PCS) and the mental component summary (MCS) scores had ES=0.31. Men and women had similar scores. Primary and secondary prevention patients scored similarly, except for worse general health in primary prevention (p=0.016, ES=0.35). Atrial fibrillation was associated with worse physical functioning (ES=0.41) and PCS (ES=0.38). Appropriate therapy, inappropriate shock, or complications requiring surgery were not associated with lower scores in any domain. In primary prevention due to ischemic vs nonischemic cardiomyopathy no domain was significantly different. PCS decreased with higher age strata (p=0.002) in contrast to MCS (p=0.986).

Conclusion Patients with ICDs have lower physical and mental HRQL than age- and sex-matched norms; however, the effect sizes are small. HRQL is similar regardless of sex, primary/secondary prevention indication, appropriate therapy, inappropriate shock, or complications, but decreases with advancing age.

For peer review only

Strengths and limitations of this study

• Real-world data on health-related quality of life in unselected ICD patients.

• Long-term ICD treatment, with associations of ICD outcomes and subgroup analyses.

• Generic health-related quality of life using SF-36 compared to age- and sex- matched population norms.

• Cross-sectional data without longitudinal assessment of health-related quality of life.

• Lack of ICD-specific patient-reported questions.

For peer review only

INTRODUCTION

An implantable cardioverter defibrillator (ICD) effectively terminates ventricular arrhythmias and protects from severe bradycardia. The ICD is a cornerstone in the treatment of heart failure with reduced ejection fraction but is also used for miscellaneous other conditions and is generally cost effective.[1–3] In addition to long-term protection from sudden cardiac death, ICDs can reduce symptoms of heart failure and prolong life when combined with cardiac resynchronization therapy (CRT).[2] In survivors of ventricular fibrillation or sustained ventricular arrhythmias with hemodynamic compromise, secondary prevention is an established indication, providing a reasonable life expectancy with good functional status for more than one year.[1,4] Using similar reasoning regarding life expectancy, primary prevention in high-risk patients is recommended.[1,4] Unfortunately, adverse events related to ICDs occur; the cumulative incidence at 12 years is about 20% inappropriate shock, 6% device-related infections, and 17% lead failure, and the incidence of complications seems to be underestimated in reports from registries.[5,6] The ICD affects several aspects of life, both professional life and leisure time activities. There may be restrictions related to driving, sports activities, and there is a risk for electromagnetic interference. ICD treatment has been associated with sleep disturbances, body-perception concerns, anxiety, and depression.

Patient-reported outcome measurements, such as health-related quality of life (HRQL), is recognized as a valuable research tool. It contributes information beyond echocardiographically assessed ejection fraction, biomarkers, or physician-assessed New York Heart Association (NYHA) functional class.[7,8] Several studies, mostly from controlled trials with interventions, have evaluated HRQL in ICD cohorts. The assessment of HRQL can be measured using a generic and/or a disease-specific questionnaire. The widely used short form (SF-36) often provides a basis for generic evaluation in diverse populations.[9,10] Whereas

For peer review only

worsening HRQL has been shown in numerous ICD cohorts, it remains largely unknown if this can be generalized into unselected ICD cohorts with long-term follow-up outside of a clinical trial.[11,12] Therefore, the primary aim of this study was to determine SF-36 scores in unselected ICD patients in comparison with age- and sex-matched general Swedish population norms. The secondary aims were to assess HRQL with regard to baseline atrial fibrillation (AF) or heart failure, appropriate ICD therapy, inappropriate ICD shock, device-related complications, and compare secondary versus primary indications.

METHODS

Design, setting, and selection

This retrospective observational study covered adult patients in Region Gävleborg, Sweden, who had an ICD implanted or underwent device replacement between 1st January 2007 and 1st January 2017. Eligible patients were identified through Provisio™, a software used for scheduling surgeries, with complete coverage of all device implants. Results from this cohort has been published previously and cover long-term follow-up with regard to appropriate therapy, complications, and mortality.[13]

Data were retrieved from electronic medical records (Melior™, Cerner Sverige AB, Stockholm) between March 2017 and February 2018 and evaluated according to a predefined protocol and imported from Excel™ 2010 (Microsoft Corporation, Redmond, WA) into SPSS™ version 22 (IBM, Armonk, NY) and Stata™ (StataCorp (2017), Stata Statistical Software: Release 15, College Station, TX) for statistical analyses. The questionnaires were distributed by regular mail, including two reminders, during 2019.

Ethics and dissemination

For peer review only

The study complies with the Declaration of Helsinki and has been approved by the Ethical Review Board in Uppsala (document number 2018/416).

Statistical analyses

Data were described as frequencies, percentages, means including standard deviations (SD), and 95% confidence intervals (CIs) were used. The t-test was used for comparisons of continuous variables. Differences in SF-36 domains between groups were tested from the non-parametric Mann-Whitney U-test. The associations between age and HQRL were tested using Spearman's non-parametric correlation coefficient for analyses and Pearson´s correlation analysis to confirm results. Age was categorized into the four strata: 32-59 years, 60-69 years, 70-79 years, and ≥80 years and differences between age strata were analyzed using the Kruskal-Wallis non-parametric analysis of variance. The estimation of the magnitude of difference between group was further assessed by calculating effect sizes (ESs), that is, the mean difference, divided by the pooled standard deviation (Cohen's *d*). The interpretation of ES was described according to standard criteria: trivial (<0.20), small (0.20-0.49), moderate (0.50-0.79), and large (≥0.80).[14] All two-sided *p*-values <0.05 were considered statistically significant. The software programs Excel 2010 (Microsoft Corporation, Redmond, WA), IBM SPSS Statistics for Windows, Version 22.0. Armonk, NY: IBM Corp., and SAS version 9.2 (SAS Institute Inc., Cary, NC) was used for analyses.

Definitions of variables

The baseline clinical characteristics of the patients were retrieved from medical records by physicians under supervision of an expert in the field (PM).

ICD therapy was defined as appropriate if an event of either antitachycardia pacing or capacitor discharge of 30-45 Joules due to an episode of ventricular arrhythmia above the

For peer review only

programmed detection interval and duration. ICD shock was classified as inappropriate if it was due to false detection or interpretation of myocardial signals (supraventricular tachycardia, T-wave oversensing) or signals outside the heart (caused by lead defect, myopotentials, electromagnetic interference).

Primary indication refers to patients who underwent ICD implant based on risk factors without any known *secondary* indication, i.e. survival of documented sustained ventricular arrhythmia with hemodynamic compromise including ventricular fibrillation.

AF or flutter had to be at least 30 seconds in duration and documented by 12-lead ECG. HF classification was based on systolic dysfunction with an ejection fraction ≤50%. The concept *complication* was restricted to events that required a surgical intervention related to the device system or the implant procedure, but excluded elective device replacement.

SF-36 domains and validation

The HRQL was assessed by the SF-36 health survey version 1.[15] This is a patient-reported multidimensional HRQL tool derived from the Medical Outcome Study in the 1990s.[10] It has been widely used for determination of the patient-perceived burden of different medical conditions and is considered a generic instrument. The instrument has 36 items, which measures eight domains that cover both physical and mental health domains: physical functioning (PF), role-physical (RP), bodily pain (BP), general health (GH), vitality (VT), social functioning (SF), role-emotional (RE), and mental health (MH). In each domain, the scores range from 0 to 100 and higher values indicate better HRQL. Based on these domains, the physical component summary (PCS) and the mental component summary (MCS) can be calculated using norm-based scoring with a mean of 50. A score above 50 would be interpreted as better HRQL than the general Swedish population. The validation of the

For peer review only

Swedish SF-36 yielded Cronbach's α between 0.79 (role-emotional) and 0.93 (bodily pain) for the domains.[10]

The SF-36 profile in the study group was compared to a general population sample which was randomly selected from the Swedish SF-36 normative database (n=8,930; response rate 68%).[15] The normative sample (validated in Sweden 1991-92) was matched on sex and age and comprised 171 persons (141 males) with a mean age of 68.8 years (SD 11.1).

Patient and public involvement

Patients were not involved in the design, or conduct, or reporting, or dissemination plan of our research.

Results

Cohort characteristics and follow-up

The SF-36 questionnaire was returned by 223 ICD patients which corresponds to a response rate of 77.2%. The mean age was similar between those who responded and those who did not (p=0.276). The same was seen between sexes (p=0.255). Figure 1 depicts the flow-chart of the emerged cohort. The majority was men 82.1% (n=183) and 60.1% (n=134) had an ICD due to primary prevention. All leads in the device systems were implanted transvenously. The baseline characteristics of the patients are summarized in Table 1.

A total of 52 patients (23.3%) experienced appropriate ICD therapy, whereas 14 (6.3%) experienced inappropriate ICD shock during the follow-up period. Complications which required surgical intervention occurred in 39 patients (17.5%). Among patients with cardiomyopathy, 76 had ischemic disease and 56 had no known underlying ischemic disease.

For peer review only

The ages ranged from 32 to 90 years with a median age of 71.2 years. The mean age was 71.1 SD 9.7 years. Men and women had similar mean ages (71.1 vs 70.9 years). Similarly, the mean ages were not different for primary vs secondary prevention (70.3 vs 72.3 years; p=0.138), a history of appropriate ICD therapy (70.7 vs 72.3 years; p=0.286), inappropriate shock (71.1 vs 71.0 years; p=0.967), or a complication requiring surgery (71.1 vs 70.9 years; p=0.907). On the contrary, patients with ischemic cardiomyopathy were older than nonischemic cardiomyopathy patients (72.9 vs 66.9 years; p<0.001) as were patients with a history of AF at baseline (73.4 vs 70.1 years; p=0.024).

HRQL in the ICD cohort compared to general population norms

In six out of eight SF-36 domains, there was a significantly lower score in ICD patients compared to sex- and aged-matched population norms (Table 2 and Figure 2).

There was a statistically significant (p<0.05) difference for PF, RP, GH, VT, SF, and MH, but not for BP and RE. The effect size among the significantly lower domains ranged from 0.23 (RP) to 0.41 (GH). Both component scores were significantly lower and the effect sizes similar, PCS (0.31) and MCS (0.31). Thus, all effect sizes were classified as small. Men and women reported similar SF-36 scores with nonsignificant differences in all domains including the component scores; PCS 41.0 SD 12.4 vs 39.5 SD 12.0 (p=0.406) and MCS 48.7 SD 10.9 vs 48.3 SD 10.5 (p=0.644)

Increasing age was associated with significantly lower scores on PF (r=-0.36; p<0.001), RP (r=-0.25; p<0.001), BP (r=-0.20; p=0.003), RE (r=-0.16; p=0.017), and PCS (r=-0.28; p<0.001).

SF-36 scores for the following age strata: 32-59, 60-69, 70-79, and ≥80 years, are reported in Table 3. Kruskal-Wallis test showed significantly lower scores for PF, RP, and PCS, while BP and RE showed a trend toward lower scores.

For peer review only

HRQL subgroup analyses

The subgroup analyses for AF, complications, appropriate therapy, and inappropriate shocks are reported in Table 4. Patients with primary-prevention ICDs were compared to secondary prevention indications. The age was similar, 70.3 vs 72.3 years. Primary-prevention patients scored lower GH (p=0.016; ES 0.35) and there was a trend toward lower VT (p=0.097; ES=0.21), but otherwise no significant differences.

A history of AF at baseline was associated with significantly lower PF (p=0.004; ES=0.41) and a trend toward lower BP (p=0.083; ES=0.27) and VT (p=0.057; ES=0.31). This translated into lower PCS (p=0.013; ES 0.38) but similar MCS (p=0.928). However, the mean age of patients with AF was slightly higher (73.4 vs 70.1 years; p=0.024).

The mean age of patients who experienced appropriate ICD therapy patients was similar to patients who did not receive appropriate shocks (72.3 vs 70.7 years) and all SF-36 domains were similar without any significant differences or trends. The group who experienced inappropriate ICD therapy was small, but had a similar mean age and no significant differences or trends with regard to any SF-36 domain. Furthermore, the group who required surgical intervention due to a complication had a similar mean age and SF-36 scores without significant differences.

In the primary-prevention group, nonischemic vs ischemic cardiomyopathy were compared. Notably, patients with an ischemic etiology were older (72.9 vs 66.9 years) but no SF-36 domain showed significantly different scores (RP showed a trend of lower score in ischemic cardiomyopathy, p=0.067; ES=0.31).

DISCUSSION

For peer review only

This Swedish cohort of general ICD patients report worse HRQL than age- and sex-matched population norms. Except for BP and RE, all domains were significantly lower although effect sizes were considered small. The component score, PCS and MCS, showed an equally small effect size.

Similar component score

In a recently published Danish study based on unselected ICD recipients, PCS (41.1 SD 10.3) and MCS (45.7 SD 10.8) at a mean follow-up of 7 years, were similar to our findings; poor PF, RP, GH, and VT were associated with higher long-term mortality.[16] Early randomized controlled trials with shorter follow-up (1-3 years) reported similar findings.[17,18] In the MADIT II , PCS was associated with an 89% higher mortality, while MCS showed 39% higher mortality.[19] US data from the INTRINSIC RV trial found improvement of SF-score over the first year and at 12 months, PCS was 42.2 and MCS 51.1, which is also similar to our results.[20]

Heart failure and HRQL

Systolic heart failure was the ICD indication for the vast majority of patients in our cohort, a third of whom received a CRT-D device. Several studies have determined that heart failure is a main determinant of poor HRQL.[21]

In a systematic review reviewing many studies based on SF-36 poor physical health status predicted adverse coronary artery disease and HF prognosis.[21] Using both disease-specific and generic test instruments, HF studies clearly revealed poor HRQL, but poor mental status had little impact. Moreover, follow-up time did not influence the predictive value of health status. In another review, those who reported poor disease-specific health status, in particular HF patients, had increased mortality (hazard ratio 1.39) compared with those with moderate/good health status.[11] Systematic reviews in this field are hampered by severe

For peer review only

heterogeneity across populations, different follow-up times, and diverse outcome measurements. Still, HF has repeatedly been shown to be a major determinant of poor health status.[12,22] Underlying cardiac disease and comorbidities influence HRQL and mortality.[12,23] A Cochrane review of nonischemic HF concluded: "using ICD therapy probably has little or no effect on quality of life."[24] This view is further strengthened by the fact that various ICD patients without structural heart disease report similar SF-36 scores as the general populations.[25,26]

Atrial fibrillation

A systematic review of several studies demonstrated poorer HRQL in AF patients than healthy controls or general populations.[27] Notably, studies on AF and HRQL are diverse and often from specific cohorts as part of an evaluation of an intervention. Such studies are prone to selection bias because they likely reflect highly symptomatic patients. Nevertheless, more general AF patients likewise show lower HRQL.[28,29] With a qualitative exploratory approach, the various symptoms have been further elucidated.[30] AF patients consist of subgroups including those with paroxysmal, persistent, and permanent AF; and even within groups, the burden of symptoms may vary substantially. AF is common in patients with heart failure and thus prevalent in ICD cohorts. In our study, the comparison among those with/without a history of AF at baseline showed that the association of AF with poor HRQL was more pronounced for the physical domains, but this may be at least partly explained by older age.

Age

As expected, age correlated with lower HQRL scores on physical domains. The four age strata showed that PCS deteriorated with older age and demonstrated significantly lower

For peer review only - http://bmjopen.bmj.com/site/about/guidelines.xhtml

scores in the analyses. This is likely due to the burden of heart failure, AF, and other comorbidities. Interestingly, mental health domains were not affected and remained consistent throughout the age strata. It can be speculated that patients selected for ICD therapy reflect a group with relatively good mental health status.

Primary versus secondary indication

The proportion of primary prophylactic ICDs was low. This reflect a historical view on indication and possibly a more conservative approach towards primary prevention. Overall, the HRQL was similar and solely the GH domain was significantly lower in the primary prevention group. The coping strategy may differ between primary and secondary prevention patients. A survivor of a life-threatening arrhythmia may feel gratitude but be prone to anxiety and depression. A lower SF-36 score (all domains except BP) in primary vs secondary prevention has been shown, which underlines the vulnerability in the primary-prevention group.[31] The differences may also be attributed to baseline characteristics; HF is a decisive factor for ICD implantation and translates into worse HRQL scores. Secondary- vs primary- prevention patients are likely to have different expectations and acceptance of ICD therapy and its related complications.

ICD shocks and complications

In the three separate subgroup analyses with regard to appropriate ICD therapy, inappropriate ICD shock, and complications requiring surgery, no significant differences in the SF-36 domains were observed. This is somewhat reassuring, at least on a group level. Data from the INTRINSIC RV trial found no significant difference in HRQL between patients with and without ICD shocks.[20]

For peer review only

In a Danish cohort, HF was associated with poor scores on all SF-36 domains, while age and ICD shocks correlated less. Notably, on depression/anxiety-specific instruments, ICD shocks were a determinant for outcome. This suggests that it is the comorbidity burden, rather than the ICD itself, that determines the patients' HRQL and despite the fact that device-related complications can be distressing to individuals, ICDs are well tolerated overall. Of course, multiple ICD shocks may translate into worse HRQL, especially anxiety.[32] Our study was unable to detect a difference between patients with ICD shocks and those without. Several explanations have been suggested for this: lower incidence of shocks, ability to cope with complications over time, generic instruments not specifically designed for ICDs, and the overall security and reassurance that comes from an ICD. Thus, it should be highlighted that careful device programming and management to avoid shocks and complications are crucial for optimal care.

Gender perspectives

The vast majority (82%) of patients in our study were men. This is in line with a review of 19 studies, in which the percentage of men ranged from 54% to 83%. We found similar SF-scores in men and women. In another study, only PF and VT were lower among women, although the difference was small, while for women, NYHA-class and Type D personality were strong determinants of poor HRQL.[23] Women may be more prone to anxiety and to score lower also on some SF-36 domains (PF, SF, MH) regardless of a history of ICD shock.[33] There needs to be an increased awareness of female candidates for ICD therapy, and our study support the fact that there are no overall sex differences with regard to generic HRQL.

Clinical perspective

For peer review only

HRQL assessment has the potential to provide insights helpful to the overall management of ICD patients, including selection of ICD candidates, and the reduction of hospitalization and mortality.[34] Patients with ICDs have poorer general HRQL than the general population and increasing age and HF are strong determinants of worse HRQL. The underlying burden of comorbidities is the main driver for low HRQL rather than the ICD itself. Concerning quality of life improvement, overall management of the cardiac disease manifestations and comorbidities is important but challenging despite advances in many therapeutic fields. In fact, according to a meta-analysis there have been slight improvements regarding physical components, but not for mental components.[35] However, a comprehensive cardiac rehabilitation program in ICD patients improved both GH and MH.[34] Even though ICD shocks and complications can be devastating for an individual, the overall perception is that an ICD is generally well tolerated and does not affect generic HRQL. Individual assessment of benefits and risks, not gender category, should be part of the risk assessment strategy.

Furthermore, complications of ICD therapy do not have a significant impact on HRQL and should not be a reason to refrain from offering an ICD to otherwise eligible patients. Nevertheless, ICD is lifelong therapy and clinicians should regularly monitor the patient’s ICD as well as quality of life as part of follow-up.

Limitations

The major strength of our study is the use of a “real-world” cohort, rather than strict inclusion/exclusion criteria used in randomized controlled trials, although this implies more heterogeneity than randomized controlled studies. The ICD patients included in our study come from a cohort of unselected patients without referral center bias. The SF-36 scores in the study group were compared with age- and sex-matched general population norms, that

For peer review only

although more than 20 years old, likely still represent current status. The observational design reflects common practice outside a clinical trial, but may hampered by lack of details in assessment of clinical profiles even though medical records were scrutinized. The proportion who responded to the survey was comparatively high, which implies less risk of differential bias.

The HRQL assessment comes from a cross-sectional cohort studied long after initial implant and likely reflects views from a more stabilized phase of life as an ICD patient. Nevertheless, the long-term impact on HRQL reported over the life of ICD patients remains unknown. We lack information about personality traits and socioeconomic factors, which may affect self-reported health. SF-36 is a well-validated questionnaire that allows for comparisons on a group level but is not specifically developed for disease-specific assessment. The study design has limitations with respect to determining causal pathways and merely show associations. Therefore, it should be interpreted with caution.

Conclusions

ICD patients have lower physical and mental HRQL, assessed by SF-36, than age- and sex-matched norms, although effect sizes are small. HRQL is similar regardless of sex, primary- or secondary-prevention indication, appropriate therapy, inappropriate shock, or complications but decreases with older age.

DECLARATIONS

Ethics: The study complies with the Declaration of Helsinki and has been approved by the Ethical Review Board in Uppsala (document number 2018/416).

Consent for publication: Not applicable.

For peer review only

Acknowledgements: The authors acknowledge editing by Jo Ann LeQuang of LeQ Medical who reviewed the manuscript for American English use. Thanks to Kristina Bergström for her help with data collection through the questionnaires. Region Gävleborg funded this research project.

Author contributions: Peter Magnusson: design, collection and interpretation of data, statistical analyses, writing the article. Gustav Mattsson: collection and interpretation of data, statistical analyses, critical revision writing the article. Marita Wallhagen: interpretation of data, critical revision. Jan Karlsson: statistical analyses, interpretation of data, critical revision. All authors approved the manuscript for submission.

Funding: This study had no external funding.

Competing interests: Peter Magnusson has received speaker fees or grants from Abbott, Alnylam, Amicus Therapeutics, Bayer, AstraZeneca, BMS, Boehringer-Ingelheim, Internetmedicin, Lilly, MSD, Novo Nordisk, Octopus Medical, Pfizer, Vifor Pharma, and Zoll.

Gustav Mattsson has received speaker fee from Alnylam, Internetmedicin, and MSD.

Marita Wallhagen and Jan Karlsson disclose no conflict of interest.

No external financial support or funding was received for this study.

Patient consent for publication: Not applicable.

Ethics approval: Approved by the Ethical Review Board in Uppsala (document number 2018/416)

Data sharing statement: All relevant data supporting the conclusions of this article is included within the article.

For peer review only

ORCID: Peter Magnusson: https://orcid.org/0000-0001-7906-7782

Gustav Mattsson: https://orcid.org/0000-0002-4317-0443

Marita Wallhagen: https://orcid.org/0000-0001-8413-3975

References

1. Priori SG, Blomström-Lundqvist C, Mazzanti A, et al. 2015 ESC Guidelines for the management of patients with ventricular arrhythmias and the prevention of sudden cardiac death: The Task Force for the Management of Patients with Ventricular Arrhythmias and the Prevention of Sudden Cardiac Death of the European Society of Cardiology (ESC). Endorsed by: Association for European Paediatric and Congenital Cardiology (AEPC). Eur Heart J 2015;36(41):2793–867.

2. Goldenberg I, Gillespie J, Moss AJ, et al. Long-term benefit of primary prevention with an implantable cardioverter-defibrillator: an extended 8-year follow-up study of the Multicenter Automatic Defibrillator Implantation Trial II. Circulation 2010;122(13):1265–71.

3. Smith T, Jordaens L, Theuns DAMJ, van Dessel PF, Wilde AA, Hunink MGM. The cost-effectiveness of primary prophylactic implantable defibrillator therapy in patients with ischaemic or non-ischaemic heart disease: a European analysis. Eur Heart J 2013;34(3):211–9.

4. Al-Khatib SM, Stevenson WG, Ackerman MJ, et al. 2017 AHA/ACC/HRS Guideline for Management of Patients With Ventricular Arrhythmias and the Prevention of Sudden Cardiac Death: A Report of the American College of Cardiology/American Heart Association Task Force on Clinical Practice Guidelines and the Heart Rhythm Society. J Am Coll Cardiol 2018;72(14):e91–220.

5. van der Heijden AC, Borleffs CJW, Buiten MS, et al. The clinical course of patients with implantable cardioverter-defibrillators: Extended experience on clinical outcome, device replacements, and device-related complications. Heart Rhythm 2015;12(6):1169–76.

6. Kirkfeldt RE, Johansen JB, Nohr EA, Jørgensen OD, Nielsen JC. Complications after cardiac implantable electronic device implantations: an analysis of a complete, nationwide cohort in Denmark. Eur Heart J 2014;35(18):1186–94.

7. Rector TS, Anand IS, Cohn JN. Relationships between clinical assessments and patients' perceptions of the effects of heart failure on their quality of life. J Card Fail 2006;12(2):87–92.

For peer review only

8. Luther SA, McCullough PA, Havranek EP, et al. The relationship between B-type natriuretic peptide and health status in patients with heart failure. J Card Fail 2005;11(6):414–21.

9. McHorney CA, Ware JE, Raczek AE. The MOS 36-Item Short-Form Health Survey (SF-36): II. Psychometric and clinical tests of validity in measuring physical and mental health constructs. Med Care 1993;31(3):247–63.

10. McHorney CA, Ware JE, Lu JF, Sherbourne CD. The MOS 36-item Short-Form Health Survey (SF-36): III. Tests of data quality, scaling assumptions, and reliability across diverse patient groups. Med Care 1994;32(1):40–66.

11. Mastenbroek MH, Versteeg H, Zijlstra W, Meine M, Spertus JA, Pedersen SS. Disease-specific health status as a predictor of mortality in patients with heart failure: a systematic literature review and meta-analysis of prospective cohort studies. Eur J Heart Fail 2014;16(4):384–93.

12. Hoogwegt MT, Kupper N, Jordaens L, Pedersen SS, Theuns DAMJ. Comorbidity burden is associated with poor psychological well-being and physical health status in patients with an implantable cardioverter-defibrillator. Europace 2013;15(10):1468–74.

13. Mattsson G, Magnusson P. Long-term follow-up of implantable cardioverter defibrillator patients with regard to appropriate therapy, complications, and mortality. Pacing Clin Electrophysiol 2020;43(2):245–53.

14. Cohen J. Statistical power analysis for the behavioral sciences. 2nd ed. Hillsdale, N.J: L. Erlbaum Associates; 1988.

15. Sullivan M, Karlsson J, Ware JE. The Swedish SF-36 Health Survey--I. Evaluation of data quality, scaling assumptions, reliability and construct validity across general populations in Sweden. Soc Sci Med 1995;41(10):1349–58.

16. van Veen B, Andersen CM, Johansen JB, Theuns DA, Pedersen SS. Patient-Reported Quality of Life as a Predictor of Mortality and Ventricular Tachyarrhythmia's During 7 Years' Follow-Up in Patients With an Implantable Cardioverter Defibrillator (from the MIDAS Study). Am J Cardiol 2019;123(4):605–10.

17. Steinberg JS, Joshi S, Schron EB, et al. Psychosocial status predicts mortality in patients with life-threatening ventricular arrhythmias. Heart Rhythm 2008;5(3):361–5.

18. Kao C-W, Friedmann E, Thomas SA. Quality of life predicts one-year survival in patients with implantable cardioverter defibrillators. Qual Life Res 2010;19(3):307–15.

19. Piotrowicz K, Noyes K, Lyness JM, et al. Physical functioning and mental well-being in association with health outcome in patients enrolled in the Multicenter Automatic Defibrillator Implantation Trial II. Eur Heart J 2007;28(5):601–7.

20. Gopinathannair R, Lerew DR, Cross NJ, Sears SF, Brown S, Olshansky B. Longitudinal changes in quality of life following ICD implant and the impact of age, gender, and ICD

For peer review only

shocks: observations from the INTRINSIC RV trial. J Interv Card Electrophysiol 2017;48(3):291–8.

21. Mommersteeg PMC, Denollet J, Spertus JA, Pedersen SS. Health status as a risk factor in cardiovascular disease: a systematic review of current evidence. Am Heart J 2009;157(2):208–18.

22. Johansen JB, Pedersen SS, Spindler H, Andersen K, Nielsen JC, Mortensen PT. Symptomatic heart failure is the most important clinical correlate of impaired quality of life, anxiety, and depression in implantable cardioverter-defibrillator patients: a single-centre, cross-sectional study in 610 patients. Europace 2008;10(5):545–51.

23. Habibović M, van den Broek KC, Theuns DAMJ, et al. Gender disparities in anxiety and quality of life in patients with an implantable cardioverter-defibrillator. Europace 2011;13(12):1723–30.

24. El Moheb M, Nicolas J, Khamis AM, Iskandarani G, Akl EA, Refaat M. Implantable cardiac defibrillators for people with non-ischaemic cardiomyopathy. Cochrane Database Syst Rev 2018;12:CD012738.

25. Probst V, Plassard-Kerdoncuf D, Mansourati J, et al. The psychological impact of implantable cardioverter defibrillator implantation on Brugada syndrome patients. Europace 2011;13(7):1034–9.

26. Christiaans I, van Langen IM, Birnie E, Bonsel GJ, Wilde AAM, Smets EMA. Quality of life and psychological distress in hypertrophic cardiomyopathy mutation carriers: a cross-sectional cohort study. Am J Med Genet A 2009;149A(4):602–12.

27. Thrall G, Lane D, Carroll D, Lip GYH. Quality of life in patients with atrial fibrillation: a systematic review. Am J Med 2006;119(5):448.e1-19.

28. Dorian P, Jung W, Newman D, et al. The impairment of health-related quality of life in patients with intermittent atrial fibrillation: implications for the assessment of investigational therapy. J Am Coll Cardiol 2000;36(4):1303–9.

29. van den Berg MP, Hassink RJ, Tuinenburg AE, et al. Quality of life in patients with paroxysmal atrial fibrillation and its predictors: importance of the autonomic nervous system. Eur Heart J 2001;22(3):247–53.

30. Stridsman M, Strömberg A, Hendriks J, Walfridsson U. Patients' Experiences of Living with Atrial Fibrillation: A Mixed Methods Study. Cardiol Res Pract 2019;2019:6590358.

31. Kikkenborg Berg S, Caspar Thygesen L, Hastrup Svendsen J, Vinggaard Christensen A, Zwisler A-D. Anxiety predicts mortality in ICD patients: results from the cross-sectional national CopenHeartICD survey with register follow-up. Pacing Clin Electrophysiol 2014;37(12):1641–50.

32. Redhead AP, Turkington D, Rao S, Tynan MM, Bourke JP. Psychopathology in postinfarction patients implanted with cardioverter-defibrillators for secondary

For peer review only

prevention. A cross-sectional, case-controlled study. J Psychosom Res 2010;69(6):555–63.

33. Spindler H, Johansen JB, Andersen K, Mortensen P, Pedersen SS. Gender differences in anxiety and concerns about the cardioverter defibrillator. Pacing Clin Electrophysiol 2009;32(5):614–21.

34. Kosiborod M, Soto GE, Jones PG, et al. Identifying heart failure patients at high risk for near-term cardiovascular events with serial health status assessments. Circulation 2007;115(15):1975–81.

35. Kao C-W, Chen M-Y, Chen T-Y, Lin P-H. Effect of psycho-educational interventions on quality of life in patients with implantable cardioverter defibrillators: a meta-analysis of randomized controlled trials. Health Qual Life Outcomes 2016;14(1):138.

For peer review only

● **Table 1.** Characteristics at ICD implant of 223 patients who completed the SF-36.

Patients	223
Mean age at implant	64.1 ± 10.9 years
Mean age at SF-36	71.1 ± 9.7 years
Females	40 (17.9%)
Device type	
ICD-VR	36 (16.1%)
ICD-DR	112 (50.2%)
CRT-D	75 (33.6%)
Hypertension	104 (46.6%)
Diabetes mellitus	47 (21.1%)
Renal failure*	27 (12.1%)
Atrial fibrillation	62 (27.8%)
Beta-blockers	201 (90.1%)
ACE-i/ARB	193 (86.5%)
MRA	104 (46.6%)

Data presented as frequencies (percentage in parenthesis).

ACE-I, angiotensin converting enzyme inhibitor; ARB, angiotensin receptor blockers; CRT-D, cardiac resynchronization therapy defibrillator; ICD-DR, dual lead implantable cardioverter defibrillator; ICD-VR, single lead implantable cardioverter defibrillator; MRA, mineralcorticoid receptor antagonist

*defined as S-Creatinine ≥130 µmol/L

For peer review only

● **Table 2:** SF-36 score in patients with ICDs compared to general Swedish population norms.

**SF-36 domains**	**Cohort mean**	**SD**	**95% CI**	**Effect size**	**Norm mean**	**SD**	**95% CI**	***p*-value**
Physical functioning	65.1	28.1	61.4-68.8	0.38	75.5	26.0	71.5-79.6	**0.0002**
Role physical	56.9	44.1	50.9-62.8	0.23	66.7	40.4	60.2-73.2	**0.0466**
Bodily pain	71.9	29.9	67.9-75.8		71.0	27.2	66.9-75.1	0.6395
General health	56.2	21.7	53.3-59.1	0.41	65.9	25.3	61.9-70.0	**<0.0001**
Vitality	58.0	24.8	54.7-61.3	0.39	68.1	27.2	63.7-72.4	**<0.0001**
Social functioning	80.1	24.4	76.8-83.3	0.25	85.9	22.9	82.4-89.4	**0.0025**
Role emotional	72.2	39.9	66.9-77.6		76.7	35.7	71.1-82.4	0.3730
Mental health	76.2	19.4	73.6-78.8	0.38	83.8	20.9	80.5-87.2	**<0.0001**
Physical Component Summary	40.7	12.3	39.0-42.4	0.31	44.4	11.6	42.4-46.4	**0.0052**
Mental Component Summary	48.6	10.8	47.2-50.1	0.31	51.9	10.5	50.1-53.8	**0.0010**

For peer review only

● **Table 3:** SF-36 score in patients with implantable cardioverter defibrillators stratified by age.

	**32-59** (n=25)	**60-69** (n=72)	**70-79** (n=76)	**80+** (n=50)	
**SF-36 domains**	Mean (SD)	Mean (SD)	Mean (SD)	Mean (SD)	*p*-value*
**PF**	79.4 (25.4)	71.9 (26.3)	62.7 (29.0)	51.4 (24.4)	**<0.0001**
**RP**	80.0 (36.8)	60.0 (44.9)	56.0 (43.9)	40.9 (41.6)	**0.0050**
**BP**	80.8 (29.3)	74.4 (30.9)	71.5 (28.8)	64.2 (29.2)	0.0780
**GH**	57.5 (21.2)	56.0 (22.6)	56.2 (21.9)	55.7 (20.7)	0.9827
**VT**	64.6 (20.4)	59.7 (27.5)	56.5 (23.8)	54.3 (23.9)	0.2363
**SF**	84.0 (22.7)	79.3 (27.9)	80.3 (22.1)	78.8 (23.5)	0.8341
**RE**	82.7 (33.5)	75.6 (38.6)	73.6 (39.1)	59.7 (44.0)	0.0773
**MH**	77.8 (18.2)	77.2 (21.2)	74.4 (18.8)	76.8 (18.3)	0.6190
**PCS**	46.6 (11.1)	42.2 (12.7)	40.0 (11.9)	36.0 (11.4)	**0.0021**
**MCS**	48.6 (10.3)	48.3 (12.0)	48.6 (10.4)	49.3 (10.1)	0.9859

For peer review only

● **Table 4:** Subgroup analyses of ICD patients.

ES=effect size *Effect sizes were *lower* when atrial fibrillation was present.

**SF-36 domains**	**Atrial fibrillation***		**Complications**		**Appropriate therapy**		**Inappropriate shock**	
	p-value	ES	*p*-value	ES	*p*-value	ES	*p*-value	ES
Physical functioning	**0.004***	0.41	0.500		0.947		0.592	
Role physical	0.128		0.210		0.897		0.428	
Bodily pain	**0.083***	0.27	0.167		0.544		0.381	
General health	0.606		0.190		0.863		0.921	
Vitality	**0.057***	0.31	0.321		0.919		0.765	
Social functioning	0.669		0.910		0.943		0.944	
Role emotional	0.313		0.972		0.796		0.955	
Mental health	0.912		0.769		0.747		0.972	
Physical Component Summary	**0.013***	0.38	0.107		0.696		0.464	
Mental Component Summary	0.923		0.902		0.772		0.375	

For peer review only

Caption:

Figure 1. Flowchart.

Figure 2. Bar chart: Health-related quality of life among ICD patients compared to age- and sex-matched norms.

For peer review only

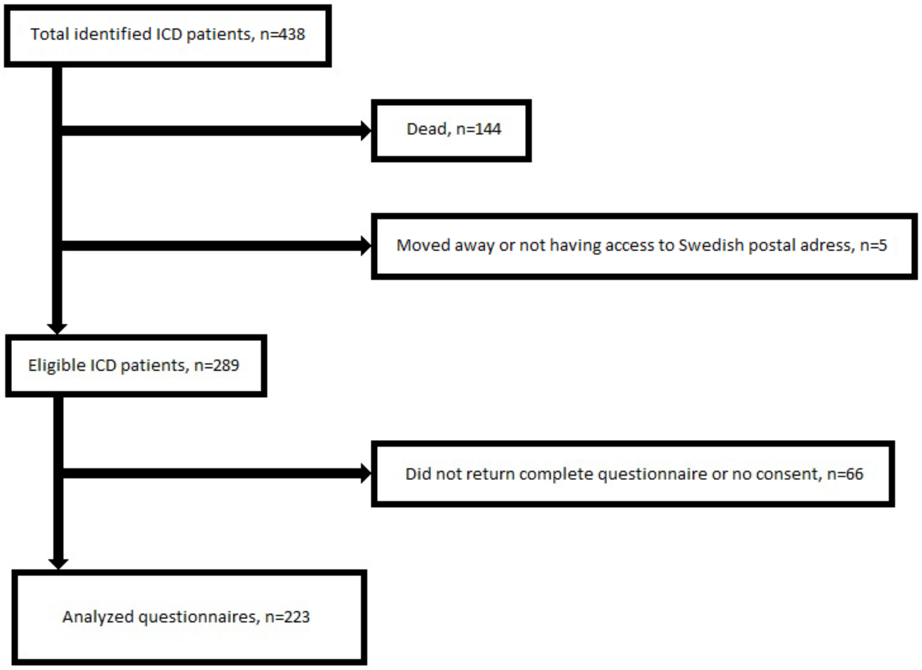

66x49mm (300 x 300 DPI)

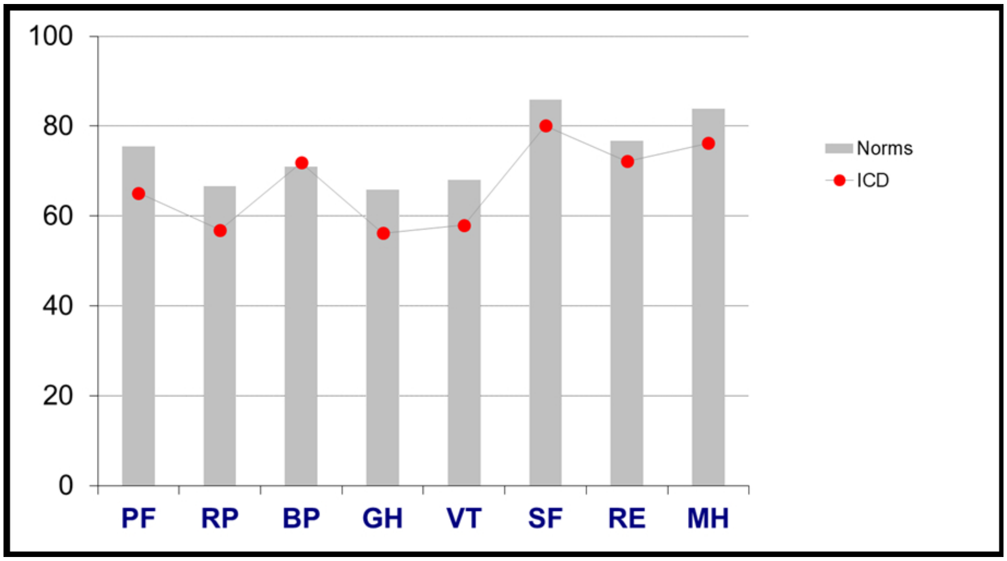
100
80
60
40
20
0
PF
RP
BP
GH
VT
SF
RE
MH
Norms
ICD

BMJ Open

Health-related quality of life in patients with implantable cardioverter defibrillators in Sweden – a cross-sectional observational trial

Journal:	*BMJ Open*
Manuscript ID	bmjopen-2020-047053.R2
Article Type:	Original research
Date Submitted by the Author:	22-May-2021
Complete List of Authors:	Magnusson, Peter; Uppsala University,<br>Mattsson, Gustav; Uppsala University<br>Wallhagen, Marita; University of Gävle<br>Karlsson, Jan; Örebro Universitet
**Primary Subject Heading**:	Cardiovascular medicine
Secondary Subject Heading:	Cardiovascular medicine
Keywords:	CARDIOLOGY, Adult cardiology < CARDIOLOGY, Heart failure < CARDIOLOGY, Pacing & electrophysiology < CARDIOLOGY

SCHOLARONE™
Manuscripts

I, the Submitting Author has the right to grant and does grant on behalf of all authors of the Work (as defined in the below author licence), an exclusive licence and/or a non-exclusive licence for contributions from authors who are: i) UK Crown employees; ii) where BMJ has agreed a CC-BY licence shall apply, and/or iii) in accordance with the terms applicable for US Federal Government officers or employees acting as part of their official duties; on a worldwide, perpetual, irrevocable, royalty-free basis to BMJ Publishing Group Ltd ("BMJ") its licensees and where the relevant Journal is co-owned by BMJ to the co-owners of the Journal, to publish the Work in this journal and any other BMJ products and to exploit all rights, as set out in our licence.

The Submitting Author accepts and understands that any supply made under these terms is made by BMJ to the Submitting Author unless you are acting as an employee on behalf of your employer or a postgraduate student of an affiliated institution which is paying any applicable article publishing charge ("APC") for Open Access articles. Where the Submitting Author wishes to make the Work available on an Open Access basis (and intends to pay the relevant APC), the terms of reuse of such Open Access shall be governed by a Creative Commons licence – details of these licences and which Creative Commons licence will apply to this Work are set out in our licence referred to above.

Other than as permitted in any relevant BMJ Author's Self Archiving Policies, I confirm this Work has not been accepted for publication elsewhere, is not being considered for publication elsewhere and does not duplicate material already published. I confirm all authors consent to publication of this Work and authorise the granting of this licence.

For peer review only

Health-related quality of life in patients with implantable cardioverter defibrillators in Sweden – a cross-sectional observational trial

Peter Magnusson[1, 2] MD PhD; Gustav Mattsson[2] MD; Marita Wallhagen[3] PhD; Jan Karlsson[4] PhD

1. Cardiology Research Unit, Department of Medicine, Karolinska Institutet, Stockholm, SE-171 76, SWEDEN
2. Centre for Research and Development, Uppsala University/Region Gävleborg, Gävle, SE- 801 87, SWEDEN
3. Department of Building Engineering, Energy Systems and Sustainability Science, University of Gävle, Gävle, SE-80176, SWEDEN
4. University Health Care Research Center, Faculty of Medicine and Health, Örebro University, SE-70182 Örebro, SWEDEN

Corresponding author:

Peter Magnusson

Cardiology Research Unit/ Department of Medicine

Karolinska Institutet, Institution of Medicine, Solna

SE-171 76 Stockholm, SWEDEN

Phone: +46(0)705 089407 Fax: +46(0)26 154255

E-mail: peter.magnusson@regiongavleborg.se

For peer review only

Keywords: cardiomyopathy, implantable cardioverter defibrillator, quality of life

Abbreviations: AF, atrial fibrillation; BP, bodily pain; CI, confidence interval; CRT, cardiac resynchronization therapy; ICD, implantable cardioverter defibrillator; GH, general health; HF, heart failure; HRQL, health related quality of life; MH, mental health; PF, physical functioning; RE, role emotional; RP, role physical; SCD, sudden cardiac death; SD, standard deviation; SF, social functioning; SF-36, short form 36; VT, vitality

Word count: 3357 (without title page, abstract, tables, figures, and references)

Short title: HRQL in ICD

Abstract

Objective Decisions regarding implantable cardioverter defibrillators (ICDs) must consider information about presumed health-related quality of life (HRQL). The purpose of the study was to assess HRQL in ICD patients and compare it to a Swedish age- and sex-matched population.

Methods SF-36 questionnaires from ICD recipients implanted 2007-2017 (response rate 77.2%) were analyzed using Mann-Whitney U-test and effect size (ES).

Results In total, 223 patients (mean age 71.1 ±9.7 years, 82.1% men) were included. In most SF-36 domains (physical functioning, role physical, general health, vitality, social functioning, and mental health), the score for ICD patients was significantly lower (ES range 0.23-0.41, i.e. small difference) than norms, except for bodily pain and role emotional. Both physical component summary (PCS) and the mental component summary (MCS) scores had ES=0.31. Men and women had similar scores. Primary and secondary prevention patients scored similarly, except for worse general health in primary prevention (p=0.016, ES=0.35). Atrial fibrillation was associated with worse physical functioning (ES=0.41) and PCS (ES=0.38). Appropriate therapy, inappropriate shock, or complications requiring surgery were not associated with lower scores in any domain. In primary prevention due to ischemic vs nonischemic cardiomyopathy no domain was significantly different. PCS decreased with higher age strata (p=0.002) in contrast to MCS (p=0.986).

Conclusion Patients with ICDs have lower physical and mental HRQL than age- and sex-matched norms; however, the effect sizes are small. HRQL is similar regardless of sex, primary/secondary prevention indication, appropriate therapy, inappropriate shock, or complications, but decreases with advancing age.

For peer review only

Strengths and limitations of this study

• Real-world data on health-related quality of life in unselected ICD patients.

• Long-term ICD treatment, with associations of ICD outcomes and subgroup analyses.

• Generic health-related quality of life using SF-36 compared to age- and sex- matched population norms.

• Cross-sectional data without longitudinal assessment of health-related quality of life.

• Lack of ICD-specific patient-reported questions.

For peer review only

INTRODUCTION

An implantable cardioverter defibrillator (ICD) effectively terminates ventricular arrhythmias and protects from severe bradycardia. The ICD is a cornerstone in the treatment of heart failure with reduced ejection fraction but is also used for miscellaneous other conditions and is generally cost effective.[1–3] In addition to long-term protection from sudden cardiac death, ICDs can reduce symptoms of heart failure and prolong life when combined with cardiac resynchronization therapy (CRT).[2] In survivors of ventricular fibrillation or sustained ventricular arrhythmias with hemodynamic compromise, secondary prevention is an established indication, providing a reasonable life expectancy with good functional status for more than one year.[1,4] Using similar reasoning regarding life expectancy, primary prevention in high-risk patients is recommended.[1,4] Unfortunately, adverse events related to ICDs occur; the cumulative incidence at 12 years is about 20% inappropriate shock, 6% device-related infections, and 17% lead failure, and the incidence of complications seems to be underestimated in reports from registries.[5,6] The ICD affects several aspects of life, both professional life and leisure time activities. There may be restrictions related to driving, sports activities, and there is a risk for electromagnetic interference. ICD treatment has been associated with sleep disturbances, body-perception concerns, anxiety, and depression.

Patient-reported outcome measurements, such as health-related quality of life (HRQL), is recognized as a valuable research tool. It contributes information beyond echocardiographically assessed ejection fraction, biomarkers, or physician-assessed New York Heart Association (NYHA) functional class.[7,8] Several studies, mostly from controlled trials with interventions, have evaluated HRQL in ICD cohorts. The assessment of HRQL can be measured using a generic and/or a disease-specific questionnaire. The widely used short form (SF-36) often provides a basis for generic evaluation in diverse populations.[9,10] Whereas

For peer review only

worsening HRQL has been shown in numerous ICD cohorts, it remains largely unknown if this can be generalized into unselected ICD cohorts with long-term follow-up outside of a clinical trial.[11,12] Therefore, the primary aim of this study was to determine SF-36 scores in unselected ICD patients in comparison with age- and sex-matched general Swedish population norms. The secondary aims were to assess HRQL with regard to baseline atrial fibrillation (AF) or heart failure, appropriate ICD therapy, inappropriate ICD shock, device-related complications, and compare secondary versus primary indications.

METHODS

Design, setting, and selection

This retrospective observational study covered adult patients in Region Gävleborg, Sweden, who had an ICD implanted or underwent device replacement between 1st January 2007 and 1st January 2017. Eligible patients were identified through Provisio™, a software used for scheduling surgeries, with complete coverage of all device implants. Results from this cohort has been published previously and cover long-term follow-up with regard to appropriate therapy, complications, and mortality.[13]

Data were retrieved from electronic medical records (Melior™, Cerner Sverige AB, Stockholm) between March 2017 and February 2018 and evaluated according to a predefined protocol and imported from Excel™ 2010 (Microsoft Corporation, Redmond, WA) into SPSS™ version 22 (IBM, Armonk, NY) and Stata™ (StataCorp (2017), Stata Statistical Software: Release 15, College Station, TX) for statistical analyses. The questionnaires were distributed by regular mail, including two reminders, during 2019.

Ethics and dissemination

For peer review only

The study complies with the Declaration of Helsinki and has been approved by the Ethical Review Board in Uppsala (document number 2018/416).

Statistical analyses

Data were described as frequencies, percentages, means including standard deviations (SD), and 95% confidence intervals (CIs) were used. The t-test was used for comparisons of continuous variables. Differences in SF-36 domains between groups were tested from the non-parametric Mann-Whitney U-test. The associations between age and HQRL were tested using Spearman's non-parametric correlation coefficient for analyses and Pearson´s correlation analysis to confirm results. Age was categorized into the four strata: 32-59 years, 60-69 years, 70-79 years, and ≥80 years and differences between age strata were analyzed using the Kruskal-Wallis non-parametric analysis of variance. The estimation of the magnitude of difference between group was further assessed by calculating effect sizes (ESs), that is, the mean difference, divided by the pooled standard deviation (Cohen's *d*). The interpretation of ES was described according to standard criteria: trivial (<0.20), small (0.20-0.49), moderate (0.50-0.79), and large (≥0.80).[14] All two-sided *p*-values <0.05 were considered statistically significant. The software programs Excel 2010 (Microsoft Corporation, Redmond, WA), IBM SPSS Statistics for Windows, Version 22.0. Armonk, NY: IBM Corp., and SAS version 9.2 (SAS Institute Inc., Cary, NC) was used for analyses.

Definitions of variables

The baseline clinical characteristics of the patients were retrieved from medical records by physicians under supervision of an expert in the field (PM).

ICD therapy was defined as appropriate if an event of either antitachycardia pacing or capacitor discharge of 30-45 Joules due to an episode of ventricular arrhythmia above the

For peer review only

programmed detection interval and duration. ICD shock was classified as inappropriate if it was due to false detection or interpretation of myocardial signals (supraventricular tachycardia, T-wave oversensing) or signals outside the heart (caused by lead defect, myopotentials, electromagnetic interference).

Primary indication refers to patients who underwent ICD implant based on risk factors without any known *secondary* indication, i.e. survival of documented sustained ventricular arrhythmia with hemodynamic compromise including ventricular fibrillation.

AF or flutter had to be at least 30 seconds in duration and documented by 12-lead ECG. HF classification was based on systolic dysfunction with an ejection fraction ≤50%. The concept *complication* was restricted to events that required a surgical intervention related to the device system or the implant procedure, but excluded elective device replacement.

SF-36 domains and validation

The HRQL was assessed by the SF-36 health survey version 1.[15] This is a patient-reported multidimensional HRQL tool derived from the Medical Outcome Study in the 1990s.[10] It has been widely used for determination of the patient-perceived burden of different medical conditions and is considered a generic instrument. The instrument has 36 items, which measures eight domains that cover both physical and mental health domains: physical functioning (PF), role-physical (RP), bodily pain (BP), general health (GH), vitality (VT), social functioning (SF), role-emotional (RE), and mental health (MH). In each domain, the scores range from 0 to 100 and higher values indicate better HRQL. Based on these domains, the physical component summary (PCS) and the mental component summary (MCS) can be calculated using norm-based scoring with a mean of 50. A score above 50 would be interpreted as better HRQL than the general Swedish population. The validation of the

For peer review only

Swedish SF-36 yielded Cronbach's α between 0.79 (role-emotional) and 0.93 (bodily pain) for the domains.[10]

The SF-36 profile in the study group was compared to a general population sample which was randomly selected from the Swedish SF-36 normative database (n=8,930; response rate 68%).[15] The normative sample (validated in Sweden 1991-92) was matched on sex and age and comprised 171 persons (141 males) with a mean age of 68.8 years (SD 11.1).

Patient and public involvement

Patients were not involved in the design, or conduct, or reporting, or dissemination plan of our research.

Results

Cohort characteristics and follow-up

The SF-36 questionnaire was returned by 223 ICD patients which corresponds to a response rate of 77.2%. The mean age was similar between those who responded and those who did not (p=0.276). The same was seen between sexes (p=0.255). Figure 1 depicts the flow-chart of the emerged cohort. The majority was men 82.1% (n=183) and 60.1% (n=134) had an ICD due to primary prevention. All leads in the device systems were implanted transvenously. The baseline characteristics of the patients are summarized in Table 1.

A total of 52 patients (23.3%) experienced appropriate ICD therapy, whereas 14 (6.3%) experienced inappropriate ICD shock during the follow-up period. Complications which required surgical intervention occurred in 39 patients (17.5%). Among patients with cardiomyopathy, 76 had ischemic disease and 56 had no known underlying ischemic disease.

For peer review only

The ages ranged from 32 to 90 years with a median age of 71.2 years. The mean age was 71.1 SD 9.7 years. Men and women had similar mean ages (71.1 vs 70.9 years). Similarly, the mean ages were not different for primary vs secondary prevention (70.3 vs 72.3 years; p=0.138), a history of appropriate ICD therapy (70.7 vs 72.3 years; p=0.286), inappropriate shock (71.1 vs 71.0 years; p=0.967), or a complication requiring surgery (71.1 vs 70.9 years; p=0.907). On the contrary, patients with ischemic cardiomyopathy were older than nonischemic cardiomyopathy patients (72.9 vs 66.9 years; p<0.001) as were patients with a history of AF at baseline (73.4 vs 70.1 years; p=0.024).

HRQL in the ICD cohort compared to general population norms

In six out of eight SF-36 domains, there was a significantly lower score in ICD patients compared to sex- and aged-matched population norms (Table 2 and Figure 2).

There was a statistically significant (p<0.05) difference for PF, RP, GH, VT, SF, and MH, but not for BP and RE. The effect size among the significantly lower domains ranged from 0.23 (RP) to 0.41 (GH). Both component scores were significantly lower and the effect sizes similar, PCS (0.31) and MCS (0.31). Thus, all effect sizes were classified as small. Men and women reported similar SF-36 scores with nonsignificant differences in all domains including the component scores; PCS 41.0 SD 12.4 vs 39.5 SD 12.0 (p=0.406) and MCS 48.7 SD 10.9 vs 48.3 SD 10.5 (p=0.644)

Increasing age was associated with significantly lower scores on PF (r=-0.36; p<0.001), RP (r=-0.25; p<0.001), BP (r=-0.20; p=0.003), RE (r=-0.16; p=0.017), and PCS (r=-0.28; p<0.001).

SF-36 scores for the following age strata: 32-59, 60-69, 70-79, and ≥80 years, are reported in Table 3. Kruskal-Wallis test showed significantly lower scores for PF, RP, and PCS, while BP and RE showed a trend toward lower scores.

For peer review only

HRQL subgroup analyses

The subgroup analyses for AF, complications, appropriate therapy, and inappropriate shocks are reported in Table 4. Patients with primary-prevention ICDs were compared to secondary prevention indications. The age was similar, 70.3 vs 72.3 years. Primary-prevention patients scored lower GH (p=0.016; ES 0.35) and there was a trend toward lower VT (p=0.097; ES=0.21), but otherwise no significant differences.

A history of AF at baseline was associated with significantly lower PF (p=0.004; ES=0.41) and a trend toward lower BP (p=0.083; ES=0.27) and VT (p=0.057; ES=0.31). This translated into lower PCS (p=0.013; ES 0.38) but similar MCS (p=0.928). However, the mean age of patients with AF was slightly higher (73.4 vs 70.1 years; p=0.024).

The mean age of patients who experienced appropriate ICD therapy patients was similar to patients who did not receive appropriate shocks (72.3 vs 70.7 years) and all SF-36 domains were similar without any significant differences or trends. The group who experienced inappropriate ICD therapy was small, but had a similar mean age and no significant differences or trends with regard to any SF-36 domain. Furthermore, the group who required surgical intervention due to a complication had a similar mean age and SF-36 scores without significant differences.

In the primary-prevention group, nonischemic vs ischemic cardiomyopathy were compared. Notably, patients with an ischemic etiology were older (72.9 vs 66.9 years) but no SF-36 domain showed significantly different scores (RP showed a trend of lower score in ischemic cardiomyopathy, p=0.067; ES=0.31).

DISCUSSION

For peer review only

This Swedish cohort of general ICD patients report worse HRQL than age- and sex-matched population norms. Except for BP and RE, all domains were significantly lower although effect sizes were considered small. The component score, PCS and MCS, showed an equally small effect size.

Similar component score

In a recently published Danish study based on unselected ICD recipients, PCS (41.1 SD 10.3) and MCS (45.7 SD 10.8) at a mean follow-up of 7 years, were similar to our findings; poor PF, RP, GH, and VT were associated with higher long-term mortality.[16] Early randomized controlled trials with shorter follow-up (1-3 years) reported similar findings.[17,18] In the MADIT II , PCS was associated with an 89% higher mortality, while MCS showed 39% higher mortality.[19] US data from the INTRINSIC RV trial found improvement of SF-score over the first year and at 12 months, PCS was 42.2 and MCS 51.1, which is also similar to our results.[20]

Heart failure and HRQL

Systolic heart failure was the ICD indication for the vast majority of patients in our cohort, a third of whom received a CRT-D device. Several studies have determined that heart failure is a main determinant of poor HRQL.[21]

In a systematic review reviewing many studies based on SF-36 poor physical health status predicted adverse coronary artery disease and HF prognosis.[21] Using both disease-specific and generic test instruments, HF studies clearly revealed poor HRQL, but poor mental status had little impact. Moreover, follow-up time did not influence the predictive value of health status. In another review, those who reported poor disease-specific health status, in particular HF patients, had increased mortality (hazard ratio 1.39) compared with those with moderate/good health status.[11] Systematic reviews in this field are hampered by severe

For peer review only

heterogeneity across populations, different follow-up times, and diverse outcome measurements. Still, HF has repeatedly been shown to be a major determinant of poor health status.[12,22] Underlying cardiac disease and comorbidities influence HRQL and mortality.[12,23] A Cochrane review of nonischemic HF concluded: "using ICD therapy probably has little or no effect on quality of life."[24] This view is further strengthened by the fact that various ICD patients without structural heart disease report similar SF-36 scores as the general populations.[25,26]

Atrial fibrillation

A systematic review of several studies demonstrated poorer HRQL in AF patients than healthy controls or general populations.[27] Notably, studies on AF and HRQL are diverse and often from specific cohorts as part of an evaluation of an intervention. Such studies are prone to selection bias because they likely reflect highly symptomatic patients. Nevertheless, more general AF patients likewise show lower HRQL.[28,29] With a qualitative exploratory approach, the various symptoms have been further elucidated.[30] AF patients consist of subgroups including those with paroxysmal, persistent, and permanent AF; and even within groups, the burden of symptoms may vary substantially. AF is common in patients with heart failure and thus prevalent in ICD cohorts. In our study, the comparison among those with/without a history of AF at baseline showed that the association of AF with poor HRQL was more pronounced for the physical domains, but this may be at least partly explained by older age.

Age

As expected, age correlated with lower HQRL scores on physical domains. The four age strata showed that PCS deteriorated with older age and demonstrated significantly lower

For peer review only

scores in the analyses. This is likely due to the burden of heart failure, AF, and other comorbidities. Interestingly, mental health domains were not affected and remained consistent throughout the age strata. It can be speculated that patients selected for ICD therapy reflect a group with relatively good mental health status.

Primary versus secondary indication

The proportion of primary prophylactic ICDs was low. This reflect a historical view on indication and possibly a more conservative approach towards primary prevention. Overall, the HRQL was similar and solely the GH domain was significantly lower in the primary prevention group. The coping strategy may differ between primary and secondary prevention patients. A survivor of a life-threatening arrhythmia may feel gratitude but be prone to anxiety and depression. A lower SF-36 score (all domains except BP) in primary vs secondary prevention has been shown, which underlines the vulnerability in the primary-prevention group.[31] The differences may also be attributed to baseline characteristics; HF is a decisive factor for ICD implantation and translates into worse HRQL scores. Secondary- vs primary- prevention patients are likely to have different expectations and acceptance of ICD therapy and its related complications.

ICD shocks and complications

In the three separate subgroup analyses with regard to appropriate ICD therapy, inappropriate ICD shock, and complications requiring surgery, no significant differences in the SF-36 domains were observed. This is somewhat reassuring, at least on a group level. Data from the INTRINSIC RV trial found no significant difference in HRQL between patients with and without ICD shocks.[20]

For peer review only

In a Danish cohort, HF was associated with poor scores on all SF-36 domains, while age and ICD shocks correlated less. Notably, on depression/anxiety-specific instruments, ICD shocks were a determinant for outcome. This suggests that it is the comorbidity burden, rather than the ICD itself, that determines the patients' HRQL and despite the fact that device-related complications can be distressing to individuals, ICDs are well tolerated overall. Of course, multiple ICD shocks may translate into worse HRQL, especially anxiety.[32] Our study was unable to detect a difference between patients with ICD shocks and those without. Several explanations have been suggested for this: lower incidence of shocks, ability to cope with complications over time, generic instruments not specifically designed for ICDs, and the overall security and reassurance that comes from an ICD. Thus, it should be highlighted that careful device programming and management to avoid shocks and complications are crucial for optimal care.

Gender perspectives

The vast majority (82%) of patients in our study were men. This is in line with a review of 19 studies, in which the percentage of men ranged from 54% to 83%. We found similar SF-scores in men and women. In another study, only PF and VT were lower among women, although the difference was small, while for women, NYHA-class and Type D personality were strong determinants of poor HRQL.[23] Women may be more prone to anxiety and to score lower also on some SF-36 domains (PF, SF, MH) regardless of a history of ICD shock.[33] There needs to be an increased awareness of female candidates for ICD therapy, and our study support the fact that there are no overall sex differences with regard to generic HRQL.

Clinical perspective

For peer review only

HRQL assessment has the potential to provide insights helpful to the overall management of ICD patients, including selection of ICD candidates, and the reduction of hospitalization and mortality.[34] Patients with ICDs have poorer general HRQL than the general population and increasing age and HF are strong determinants of worse HRQL. The underlying burden of comorbidities is the main driver for low HRQL rather than the ICD itself. Concerning quality of life improvement, overall management of the cardiac disease manifestations and comorbidities is important but challenging despite advances in many therapeutic fields. In fact, according to a meta-analysis there have been slight improvements regarding physical components, but not for mental components.[35] However, a comprehensive cardiac rehabilitation program in ICD patients improved both GH and MH.[34] Even though ICD shocks and complications can be devastating for an individual, the overall perception is that an ICD is generally well tolerated and does not affect generic HRQL. Individual assessment of benefits and risks, not gender category, should be part of the risk assessment strategy.

Furthermore, complications of ICD therapy do not have a significant impact on HRQL and should not be a reason to refrain from offering an ICD to otherwise eligible patients. Nevertheless, ICD is lifelong therapy and clinicians should regularly monitor the patient's ICD as well as quality of life as part of follow-up.

Limitations

The major strength of our study is the use of a "real-world" cohort, rather than strict inclusion/exclusion criteria used in randomized controlled trials, although this implies more heterogeneity than randomized controlled studies. The ICD patients included in our study come from a cohort of unselected patients without referral center bias. The SF-36 scores in the study group were compared with age- and sex-matched general population norms, that

For peer review only

although more than 20 years old, likely still represent current status. The observational design reflects common practice outside a clinical trial, but may hampered by lack of details in assessment of clinical profiles even though medical records were scrutinized. The proportion who responded to the survey was comparatively high, which implies less risk of differential bias.

The HRQL assessment comes from a cross-sectional cohort studied long after initial implant and likely reflects views from a more stabilized phase of life as an ICD patient. Nevertheless, the long-term impact on HRQL reported over the life of ICD patients remains unknown. We lack information about personality traits and socioeconomic factors, which may affect self-reported health. SF-36 is a well-validated questionnaire that allows for comparisons on a group level but is not specifically developed for disease-specific assessment. The study design has limitations with respect to determining causal pathways and merely show associations. Therefore, it should be interpreted with caution.

Conclusions

ICD patients have lower physical and mental HRQL, assessed by SF-36, than age- and sex-matched norms, although effect sizes are small. HRQL is similar regardless of sex, primary- or secondary-prevention indication, appropriate therapy, inappropriate shock, or complications but decreases with older age.

DECLARATIONS

Ethics: The study complies with the Declaration of Helsinki and has been approved by the Ethical Review Board in Uppsala (document number 2018/416).

Consent for publication: Not applicable.

For peer review only

Acknowledgements: The authors acknowledge editing by Jo Ann LeQuang of LeQ Medical who reviewed the manuscript for American English use. Thanks to Kristina Bergström for her help with data collection through the questionnaires. Region Gävleborg funded this research project.

Author contributions: Peter Magnusson: design, collection and interpretation of data, statistical analyses, writing the article. Gustav Mattsson: collection and interpretation of data, statistical analyses, critical revision writing the article. Marita Wallhagen: interpretation of data, critical revision. Jan Karlsson: statistical analyses, interpretation of data, critical revision. All authors approved the manuscript for submission.

Funding: This study had no external funding.

Competing interests: Peter Magnusson has received speaker fees or grants from Abbott, Alnylam, Amicus Therapeutics, Bayer, AstraZeneca, BMS, Boehringer-Ingelheim, Internetmedicin, Lilly, MSD, Novo Nordisk, Octopus Medical, Pfizer, Vifor Pharma, and Zoll.

Gustav Mattsson has received speaker fee from Alnylam, Internetmedicin, and MSD.

Marita Wallhagen and Jan Karlsson disclose no conflict of interest.

No external financial support or funding was received for this study.

Patient consent for publication: Not applicable.

Ethics approval: Approved by the Ethical Review Board in Uppsala (document number 2018/416)

Data sharing statement: All relevant data supporting the conclusions of this article is included within the article.

For peer review only

ORCID: Peter Magnusson: https://orcid.org/0000-0001-7906-7782

Gustav Mattsson: https://orcid.org/0000-0002-4317-0443

Marita Wallhagen: https://orcid.org/0000-0001-8413-3975

References

1. Priori SG, Blomström-Lundqvist C, Mazzanti A, et al. 2015 ESC Guidelines for the management of patients with ventricular arrhythmias and the prevention of sudden cardiac death: The Task Force for the Management of Patients with Ventricular Arrhythmias and the Prevention of Sudden Cardiac Death of the European Society of Cardiology (ESC). Endorsed by: Association for European Paediatric and Congenital Cardiology (AEPC). Eur Heart J 2015;36(41):2793–867.

2. Goldenberg I, Gillespie J, Moss AJ, et al. Long-term benefit of primary prevention with an implantable cardioverter-defibrillator: an extended 8-year follow-up study of the Multicenter Automatic Defibrillator Implantation Trial II. Circulation 2010;122(13):1265–71.

3. Smith T, Jordaens L, Theuns DAMJ, van Dessel PF, Wilde AA, Hunink MGM. The cost-effectiveness of primary prophylactic implantable defibrillator therapy in patients with ischaemic or non-ischaemic heart disease: a European analysis. Eur Heart J 2013;34(3):211–9.

4. Al-Khatib SM, Stevenson WG, Ackerman MJ, et al. 2017 AHA/ACC/HRS Guideline for Management of Patients With Ventricular Arrhythmias and the Prevention of Sudden Cardiac Death: A Report of the American College of Cardiology/American Heart Association Task Force on Clinical Practice Guidelines and the Heart Rhythm Society. J Am Coll Cardiol 2018;72(14):e91–220.

5. van der Heijden AC, Borleffs CJW, Buiten MS, et al. The clinical course of patients with implantable cardioverter-defibrillators: Extended experience on clinical outcome, device replacements, and device-related complications. Heart Rhythm 2015;12(6):1169–76.

6. Kirkfeldt RE, Johansen JB, Nohr EA, Jørgensen OD, Nielsen JC. Complications after cardiac implantable electronic device implantations: an analysis of a complete, nationwide cohort in Denmark. Eur Heart J 2014;35(18):1186–94.

7. Rector TS, Anand IS, Cohn JN. Relationships between clinical assessments and patients' perceptions of the effects of heart failure on their quality of life. J Card Fail 2006;12(2):87–92.

For peer review only

8. Luther SA, McCullough PA, Havranek EP, et al. The relationship between B-type natriuretic peptide and health status in patients with heart failure. J Card Fail 2005;11(6):414–21.

9. McHorney CA, Ware JE, Raczek AE. The MOS 36-Item Short-Form Health Survey (SF-36): II. Psychometric and clinical tests of validity in measuring physical and mental health constructs. Med Care 1993;31(3):247–63.

10. McHorney CA, Ware JE, Lu JF, Sherbourne CD. The MOS 36-item Short-Form Health Survey (SF-36): III. Tests of data quality, scaling assumptions, and reliability across diverse patient groups. Med Care 1994;32(1):40–66.

11. Mastenbroek MH, Versteeg H, Zijlstra W, Meine M, Spertus JA, Pedersen SS. Disease-specific health status as a predictor of mortality in patients with heart failure: a systematic literature review and meta-analysis of prospective cohort studies. Eur J Heart Fail 2014;16(4):384–93.

12. Hoogwegt MT, Kupper N, Jordaens L, Pedersen SS, Theuns DAMJ. Comorbidity burden is associated with poor psychological well-being and physical health status in patients with an implantable cardioverter-defibrillator. Europace 2013;15(10):1468–74.

13. Mattsson G, Magnusson P. Long-term follow-up of implantable cardioverter defibrillator patients with regard to appropriate therapy, complications, and mortality. Pacing Clin Electrophysiol 2020;43(2):245–53.

14. Cohen J. Statistical power analysis for the behavioral sciences. 2nd ed. Hillsdale, N.J: L. Erlbaum Associates; 1988.

15. Sullivan M, Karlsson J, Ware JE. The Swedish SF-36 Health Survey--I. Evaluation of data quality, scaling assumptions, reliability and construct validity across general populations in Sweden. Soc Sci Med 1995;41(10):1349–58.

16. van Veen B, Andersen CM, Johansen JB, Theuns DA, Pedersen SS. Patient-Reported Quality of Life as a Predictor of Mortality and Ventricular Tachyarrhythmia's During 7 Years' Follow-Up in Patients With an Implantable Cardioverter Defibrillator (from the MIDAS Study). Am J Cardiol 2019;123(4):605–10.

17. Steinberg JS, Joshi S, Schron EB, et al. Psychosocial status predicts mortality in patients with life-threatening ventricular arrhythmias. Heart Rhythm 2008;5(3):361–5.

18. Kao C-W, Friedmann E, Thomas SA. Quality of life predicts one-year survival in patients with implantable cardioverter defibrillators. Qual Life Res 2010;19(3):307–15.

19. Piotrowicz K, Noyes K, Lyness JM, et al. Physical functioning and mental well-being in association with health outcome in patients enrolled in the Multicenter Automatic Defibrillator Implantation Trial II. Eur Heart J 2007;28(5):601–7.

20. Gopinathannair R, Lerew DR, Cross NJ, Sears SF, Brown S, Olshansky B. Longitudinal changes in quality of life following ICD implant and the impact of age, gender, and ICD

For peer review only

shocks: observations from the INTRINSIC RV trial. J Interv Card Electrophysiol 2017;48(3):291–8.

21. Mommersteeg PMC, Denollet J, Spertus JA, Pedersen SS. Health status as a risk factor in cardiovascular disease: a systematic review of current evidence. Am Heart J 2009;157(2):208–18.

22. Johansen JB, Pedersen SS, Spindler H, Andersen K, Nielsen JC, Mortensen PT. Symptomatic heart failure is the most important clinical correlate of impaired quality of life, anxiety, and depression in implantable cardioverter-defibrillator patients: a single-centre, cross-sectional study in 610 patients. Europace 2008;10(5):545–51.

23. Habibović M, van den Broek KC, Theuns DAMJ, et al. Gender disparities in anxiety and quality of life in patients with an implantable cardioverter-defibrillator. Europace 2011;13(12):1723–30.

24. El Moheb M, Nicolas J, Khamis AM, Iskandarani G, Akl EA, Refaat M. Implantable cardiac defibrillators for people with non-ischaemic cardiomyopathy. Cochrane Database Syst Rev 2018;12:CD012738.

25. Probst V, Plassard-Kerdoncuf D, Mansourati J, et al. The psychological impact of implantable cardioverter defibrillator implantation on Brugada syndrome patients. Europace 2011;13(7):1034–9.

26. Christiaans I, van Langen IM, Birnie E, Bonsel GJ, Wilde AAM, Smets EMA. Quality of life and psychological distress in hypertrophic cardiomyopathy mutation carriers: a cross-sectional cohort study. Am J Med Genet A 2009;149A(4):602–12.

27. Thrall G, Lane D, Carroll D, Lip GYH. Quality of life in patients with atrial fibrillation: a systematic review. Am J Med 2006;119(5):448.e1-19.

28. Dorian P, Jung W, Newman D, et al. The impairment of health-related quality of life in patients with intermittent atrial fibrillation: implications for the assessment of investigational therapy. J Am Coll Cardiol 2000;36(4):1303–9.

29. van den Berg MP, Hassink RJ, Tuinenburg AE, et al. Quality of life in patients with paroxysmal atrial fibrillation and its predictors: importance of the autonomic nervous system. Eur Heart J 2001;22(3):247–53.

30. Stridsman M, Strömberg A, Hendriks J, Walfridsson U. Patients' Experiences of Living with Atrial Fibrillation: A Mixed Methods Study. Cardiol Res Pract 2019;2019:6590358.

31. Kikkenborg Berg S, Caspar Thygesen L, Hastrup Svendsen J, Vinggaard Christensen A, Zwisler A-D. Anxiety predicts mortality in ICD patients: results from the cross-sectional national CopenHeartICD survey with register follow-up. Pacing Clin Electrophysiol 2014;37(12):1641–50.

32. Redhead AP, Turkington D, Rao S, Tynan MM, Bourke JP. Psychopathology in postinfarction patients implanted with cardioverter-defibrillators for secondary

For peer review only

prevention. A cross-sectional, case-controlled study. J Psychosom Res 2010;69(6):555–63.

33. Spindler H, Johansen JB, Andersen K, Mortensen P, Pedersen SS. Gender differences in anxiety and concerns about the cardioverter defibrillator. Pacing Clin Electrophysiol 2009;32(5):614–21.

34. Kosiborod M, Soto GE, Jones PG, et al. Identifying heart failure patients at high risk for near-term cardiovascular events with serial health status assessments. Circulation 2007;115(15):1975–81.

35. Kao C-W, Chen M-Y, Chen T-Y, Lin P-H. Effect of psycho-educational interventions on quality of life in patients with implantable cardioverter defibrillators: a meta-analysis of randomized controlled trials. Health Qual Life Outcomes 2016;14(1):138.

For peer review only

● **Table 1.** Characteristics at ICD implant of 223 patients who completed the SF-36.

Patients	223
Mean age at implant	64.1 ± 10.9 years
Mean age at SF-36	71.1 ± 9.7 years
Females	40 (17.9%)
Device type	
ICD-VR	36 (16.1%)
ICD-DR	112 (50.2%)
CRT-D	75 (33.6%)
Hypertension	104 (46.6%)
Diabetes mellitus	47 (21.1%)
Renal failure*	27 (12.1%)
Atrial fibrillation	62 (27.8%)
Beta-blockers	201 (90.1%)
ACE-i/ARB	193 (86.5%)
MRA	104 (46.6%)

Data presented as frequencies (percentage in parenthesis).

ACE-I, angiotensin converting enzyme inhibitor; ARB, angiotensin receptor blockers; CRT-D, cardiac resynchronization therapy defibrillator; ICD-DR, dual lead implantable cardioverter defibrillator; ICD-VR, single lead implantable cardioverter defibrillator; MRA, mineralcorticoid receptor antagonist

*defined as S-Creatinine ≥130 µmol/L

For peer review only

● **Table 2:** SF-36 score in patients with ICDs compared to general Swedish population norms.

**SF-36 domains**	**Cohort mean**	**SD**	**95% CI**	**Effect size**	**Norm mean**	**SD**	**95% CI**	***p*-value**
Physical functioning	65.1	28.1	61.4-68.8	0.38	75.5	26.0	71.5-79.6	**0.0002**
Role physical	56.9	44.1	50.9-62.8	0.23	66.7	40.4	60.2-73.2	**0.0466**
Bodily pain	71.9	29.9	67.9-75.8		71.0	27.2	66.9-75.1	0.6395
General health	56.2	21.7	53.3-59.1	0.41	65.9	25.3	61.9-70.0	**<0.0001**
Vitality	58.0	24.8	54.7-61.3	0.39	68.1	27.2	63.7-72.4	**<0.0001**
Social functioning	80.1	24.4	76.8-83.3	0.25	85.9	22.9	82.4-89.4	**0.0025**
Role emotional	72.2	39.9	66.9-77.6		76.7	35.7	71.1-82.4	0.3730
Mental health	76.2	19.4	73.6-78.8	0.38	83.8	20.9	80.5-87.2	**<0.0001**
Physical Component Summary	40.7	12.3	39.0-42.4	0.31	44.4	11.6	42.4-46.4	**0.0052**
Mental Component Summary	48.6	10.8	47.2-50.1	0.31	51.9	10.5	50.1-53.8	**0.0010**

For peer review only

● **Table 3:** SF-36 score in patients with implantable cardioverter defibrillators stratified by age.

	**32-59** (n=25)	**60-69** (n=72)	**70-79** (n=76)	**80+** (n=50)	
**SF-36 domains**	Mean (SD)	Mean (SD)	Mean (SD)	Mean (SD)	*p*-value*
**PF**	79.4 (25.4)	71.9 (26.3)	62.7 (29.0)	51.4 (24.4)	**<0.0001**
**RP**	80.0 (36.8)	60.0 (44.9)	56.0 (43.9)	40.9 (41.6)	**0.0050**
**BP**	80.8 (29.3)	74.4 (30.9)	71.5 (28.8)	64.2 (29.2)	0.0780
**GH**	57.5 (21.2)	56.0 (22.6)	56.2 (21.9)	55.7 (20.7)	0.9827
**VT**	64.6 (20.4)	59.7 (27.5)	56.5 (23.8)	54.3 (23.9)	0.2363
**SF**	84.0 (22.7)	79.3 (27.9)	80.3 (22.1)	78.8 (23.5)	0.8341
**RE**	82.7 (33.5)	75.6 (38.6)	73.6 (39.1)	59.7 (44.0)	0.0773
**MH**	77.8 (18.2)	77.2 (21.2)	74.4 (18.8)	76.8 (18.3)	0.6190
**PCS**	46.6 (11.1)	42.2 (12.7)	40.0 (11.9)	36.0 (11.4)	**0.0021**
**MCS**	48.6 (10.3)	48.3 (12.0)	48.6 (10.4)	49.3 (10.1)	0.9859

For peer review only

● **Table 4:** Subgroup analyses of ICD patients.

ES=effect size *Effect sizes were *lower* when atrial fibrillation was present.

SF-36 domains	Atrial fibrillation*		Complications		Appropriate therapy		Inappropriate shock	
	p-value	ES	*p*-value	ES	*p*-value	ES	*p*-value	ES
Physical functioning	**0.004***	0.41	0.500		0.947		0.592	
Role physical	0.128		0.210		0.897		0.428	
Bodily pain	**0.083***	0.27	0.167		0.544		0.381	
General health	0.606		0.190		0.863		0.921	
Vitality	**0.057***	0.31	0.321		0.919		0.765	
Social functioning	0.669		0.910		0.943		0.944	
Role emotional	0.313		0.972		0.796		0.955	
Mental health	0.912		0.769		0.747		0.972	
Physical Component Summary	**0.013***	0.38	0.107		0.696		0.464	
Mental Component Summary	0.923		0.902		0.772		0.375	

For peer review only

Caption:

Figure 1. Flowchart.

Figure 2. Bar chart: Health-related quality of life among ICD patients compared to age- and sex-matched norms.

For peer review only

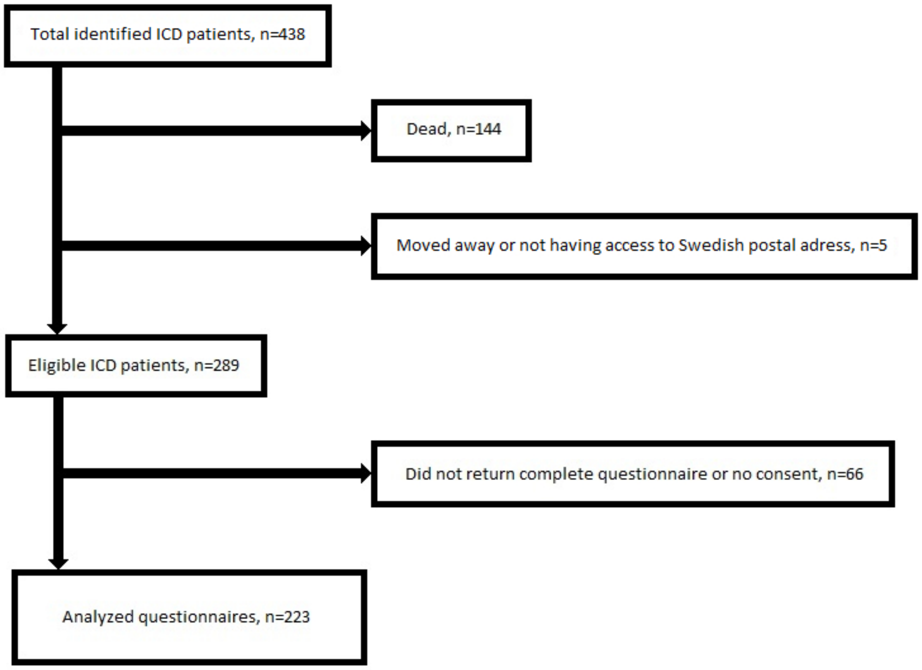

66x49mm (300 x 300 DPI)

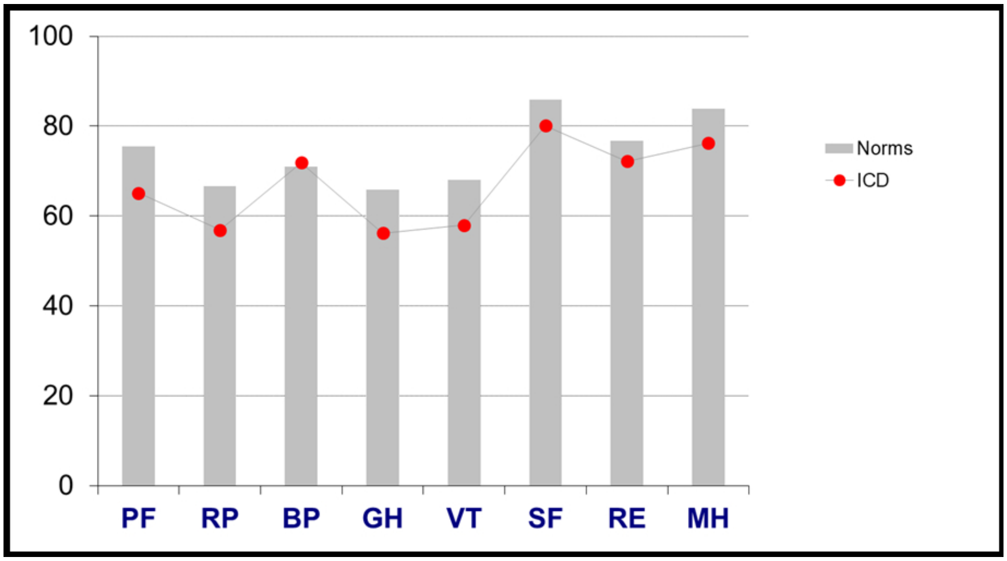
100
80
60
40
20
0
PF
RP
BP
GH
VT
SF
RE
MH
Norms
ICD

BMJ Open

Health-related quality of life in patients with implantable cardioverter defibrillators in Sweden – a cross-sectional observational trial

Journal:	*BMJ Open*
Manuscript ID	bmjopen-2020-047053.R3
Article Type:	Original research
Date Submitted by the Author:	27-May-2021
Complete List of Authors:	Magnusson, Peter; Uppsala University,<br>Mattsson, Gustav; Uppsala University<br>Wallhagen, Marita; University of Gävle<br>Karlsson, Jan; Örebro Universitet
**Primary Subject Heading**:	Cardiovascular medicine
Secondary Subject Heading:	Cardiovascular medicine
Keywords:	CARDIOLOGY, Adult cardiology < CARDIOLOGY, Heart failure < CARDIOLOGY, Pacing & electrophysiology < CARDIOLOGY

SCHOLARONE™
Manuscripts

I, the Submitting Author has the right to grant and does grant on behalf of all authors of the Work (as defined in the below author licence), an exclusive licence and/or a non-exclusive licence for contributions from authors who are: i) UK Crown employees; ii) where BMJ has agreed a CC-BY licence shall apply, and/or iii) in accordance with the terms applicable for US Federal Government officers or employees acting as part of their official duties; on a worldwide, perpetual, irrevocable, royalty-free basis to BMJ Publishing Group Ltd ("BMJ") its licensees and where the relevant Journal is co-owned by BMJ to the co-owners of the Journal, to publish the Work in this journal and any other BMJ products and to exploit all rights, as set out in our licence.

The Submitting Author accepts and understands that any supply made under these terms is made by BMJ to the Submitting Author unless you are acting as an employee on behalf of your employer or a postgraduate student of an affiliated institution which is paying any applicable article publishing charge ("APC") for Open Access articles. Where the Submitting Author wishes to make the Work available on an Open Access basis (and intends to pay the relevant APC), the terms of reuse of such Open Access shall be governed by a Creative Commons licence – details of these licences and which Creative Commons licence will apply to this Work are set out in our licence referred to above.

Other than as permitted in any relevant BMJ Author's Self Archiving Policies, I confirm this Work has not been accepted for publication elsewhere, is not being considered for publication elsewhere and does not duplicate material already published. I confirm all authors consent to publication of this Work and authorise the granting of this licence.

For peer review only

Health-related quality of life in patients with implantable cardioverter defibrillators in Sweden – a cross-sectional observational trial

Peter Magnusson[1, 2] MD PhD; Gustav Mattsson[2] MD; Marita Wallhagen[3] PhD; Jan Karlsson[4] PhD

1. Cardiology Research Unit, Department of Medicine, Karolinska Institutet, Stockholm, SE-171 76, SWEDEN
2. Centre for Research and Development, Uppsala University/Region Gävleborg, Gävle, SE- 801 87, SWEDEN

3. Department of Building Engineering, Energy Systems and Sustainability Science, University of Gävle, Gävle, SE-80176, SWEDEN

4. University Health Care Research Center, Faculty of Medicine and Health, Örebro University, SE-70182 Örebro, SWEDEN

Corresponding author:

Peter Magnusson

Cardiology Research Unit/ Department of Medicine

Karolinska Institutet, Institution of Medicine, Solna

SE-171 76 Stockholm, SWEDEN

Phone: +46(0)705 089407 Fax: +46(0)26 154255

E-mail: peter.magnusson@regiongavleborg.se

Keywords: cardiomyopathy, implantable cardioverter defibrillator, quality of life

Abbreviations: AF, atrial fibrillation; BP, bodily pain; CI, confidence interval; CRT, cardiac resynchronization therapy; ICD, implantable cardioverter defibrillator; GH, general health; HF, heart failure; HRQL, health related quality of life; MH, mental health; PF, physical functioning; RE, role emotional; RP, role physical; SCD, sudden cardiac death; SD, standard deviation; SF, social functioning; SF-36, short form 36; VT, vitality

Word count: 3357 (without title page, abstract, tables, figures, and references)

Short title: HRQL in ICD

For peer review only

Abstract

Objectives Decisions regarding implantable cardioverter defibrillators (ICDs) must consider information about presumed health-related quality of life (HRQL). The purpose of the study was to assess HRQL in ICD patients and compare it to a Swedish age- and sex-matched population.

Design cross-sectional observational trial

Setting Swedish ICD cohort

Interventions SF-36 questionnaires from ICD recipients implanted 2007-2017 (response rate 77.2%) were analyzed using Mann-Whitney U-test and effect size (ES).

Results In total, 223 patients (mean age 71.1 ±9.7 years, 82.1% men) were included. In most SF-36 domains (physical functioning, role physical, general health, vitality, social functioning, and mental health), the score for ICD patients was significantly lower (ES range 0.23-0.41, i.e. small difference) than norms, except for bodily pain and role emotional. Both physical component summary (PCS) and the mental component summary (MCS) scores had ES=0.31. Men and women had similar scores. Primary and secondary prevention patients scored similarly, except for worse general health in primary prevention (p=0.016, ES=0.35). Atrial fibrillation was associated with worse physical functioning (ES=0.41) and PCS (ES=0.38). Appropriate therapy, inappropriate shock, or complications requiring surgery were not associated with lower scores in any domain. In primary prevention due to ischemic vs nonischemic cardiomyopathy no domain was significantly different. PCS decreased with higher age strata (p=0.002) in contrast to MCS (p=0.986).

For peer review only

Conclusions Patients with ICDs have lower physical and mental HRQL than age- and sex-matched norms; however, the effect sizes are small. HRQL is similar regardless of sex, primary/secondary prevention indication, appropriate therapy, inappropriate shock, or complications, but decreases with advancing age.

Strengths and limitations of this study

- Real-world data on health-related quality of life in unselected ICD patients.
- Long-term ICD treatment, with associations of ICD outcomes and subgroup analyses.
- Generic health-related quality of life using SF-36 compared to age- and sex- matched population norms.
- Cross-sectional data without longitudinal assessment of health-related quality of life.
- Lack of ICD-specific patient-reported questions.

For peer review only

INTRODUCTION

An implantable cardioverter defibrillator (ICD) effectively terminates ventricular arrhythmias and protects from severe bradycardia. The ICD is a cornerstone in the treatment of heart failure with reduced ejection fraction but is also used for miscellaneous other conditions and is generally cost effective.[1–3] In addition to long-term protection from sudden cardiac death, ICDs can reduce symptoms of heart failure and prolong life when combined with cardiac resynchronization therapy (CRT).[2] In survivors of ventricular fibrillation or sustained ventricular arrhythmias with hemodynamic compromise, secondary prevention is an established indication, providing a reasonable life expectancy with good functional status for more than one year.[1,4] Using similar reasoning regarding life expectancy, primary prevention in high-risk patients is recommended.[1,4] Unfortunately, adverse events related to ICDs occur; the cumulative incidence at 12 years is about 20% inappropriate shock, 6% device-related infections, and 17% lead failure, and the incidence of complications seems to be underestimated in reports from registries.[5,6] The ICD affects several aspects of life, both professional life and leisure time activities. There may be restrictions related to driving, sports activities, and there is a risk for electromagnetic interference. ICD treatment has been associated with sleep disturbances, body-perception concerns, anxiety, and depression.

Patient-reported outcome measurements, such as health-related quality of life (HRQL), is recognized as a valuable research tool. It contributes information beyond echocardiographically assessed ejection fraction, biomarkers, or physician-assessed New York Heart Association (NYHA) functional class.[7,8] Several studies, mostly from controlled trials with interventions, have evaluated HRQL in ICD cohorts. The assessment of HRQL can be measured using a generic and/or a disease-specific questionnaire. The widely used short form (SF-36) often provides a basis for generic evaluation in diverse populations.[9,10] Whereas

For peer review only

worsening HRQL has been shown in numerous ICD cohorts, it remains largely unknown if this can be generalized into unselected ICD cohorts with long-term follow-up outside of a clinical trial.[11,12] Therefore, the primary aim of this study was to determine SF-36 scores in unselected ICD patients in comparison with age- and sex-matched general Swedish population norms. The secondary aims were to assess HRQL with regard to baseline atrial fibrillation (AF) or heart failure, appropriate ICD therapy, inappropriate ICD shock, device-related complications, and compare secondary versus primary indications.

METHODS

Design, setting, and selection

This retrospective observational study covered adult patients in Region Gävleborg, Sweden, who had an ICD implanted or underwent device replacement between 1st January 2007 and 1st January 2017. Eligible patients were identified through Provisio™, a software used for scheduling surgeries, with complete coverage of all device implants. Results from this cohort has been published previously and cover long-term follow-up with regard to appropriate therapy, complications, and mortality.[13]

Data were retrieved from electronic medical records (Melior™, Cerner Sverige AB, Stockholm) between March 2017 and February 2018 and evaluated according to a predefined protocol and imported from Excel™ 2010 (Microsoft Corporation, Redmond, WA) into SPSS™ version 22 (IBM, Armonk, NY) and Stata™ (StataCorp (2017), Stata Statistical Software: Release 15, College Station, TX) for statistical analyses. The questionnaires were distributed by regular mail, including two reminders, during 2019.

Ethics and dissemination

For peer review only

The study complies with the Declaration of Helsinki and has been approved by the Ethical Review Board in Uppsala (document number 2018/416).

Statistical analyses

Data were described as frequencies, percentages, means including standard deviations (SD), and 95% confidence intervals (CIs) were used. The t-test was used for comparisons of continuous variables. Differences in SF-36 domains between groups were tested from the non-parametric Mann-Whitney U-test. The associations between age and HQRL were tested using Spearman's non-parametric correlation coefficient for analyses and Pearson´s correlation analysis to confirm results. Age was categorized into the four strata: 32-59 years, 60-69 years, 70-79 years, and ≥80 years and differences between age strata were analyzed using the Kruskal-Wallis non-parametric analysis of variance. The estimation of the magnitude of difference between group was further assessed by calculating effect sizes (ESs), that is, the mean difference, divided by the pooled standard deviation (Cohen's d). The interpretation of ES was described according to standard criteria: trivial (<0.20), small (0.20-0.49), moderate (0.50-0.79), and large (≥0.80).[14] All two-sided p-values <0.05 were considered statistically significant. The software programs Excel 2010 (Microsoft Corporation, Redmond, WA), IBM SPSS Statistics for Windows, Version 22.0. Armonk, NY: IBM Corp., and SAS version 9.2 (SAS Institute Inc., Cary, NC) was used for analyses.

Definitions of variables

The baseline clinical characteristics of the patients were retrieved from medical records by physicians under supervision of an expert in the field (PM).

ICD therapy was defined as appropriate if an event of either antitachycardia pacing or capacitor discharge of 30-45 Joules due to an episode of ventricular arrhythmia above the

For peer review only

programmed detection interval and duration. ICD shock was classified as inappropriate if it was due to false detection or interpretation of myocardial signals (supraventricular tachycardia, T-wave oversensing) or signals outside the heart (caused by lead defect, myopotentials, electromagnetic interference).

Primary indication refers to patients who underwent ICD implant based on risk factors without any known *secondary* indication, i.e. survival of documented sustained ventricular arrhythmia with hemodynamic compromise including ventricular fibrillation.

AF or flutter had to be at least 30 seconds in duration and documented by 12-lead ECG. HF classification was based on systolic dysfunction with an ejection fraction ≤50%. The concept *complication* was restricted to events that required a surgical intervention related to the device system or the implant procedure, but excluded elective device replacement.

SF-36 domains and validation

The HRQL was assessed by the SF-36 health survey version 1.[15] This is a patient-reported multidimensional HRQL tool derived from the Medical Outcome Study in the 1990s.[10] It has been widely used for determination of the patient-perceived burden of different medical conditions and is considered a generic instrument. The instrument has 36 items, which measures eight domains that cover both physical and mental health domains: physical functioning (PF), role-physical (RP), bodily pain (BP), general health (GH), vitality (VT), social functioning (SF), role-emotional (RE), and mental health (MH). In each domain, the scores range from 0 to 100 and higher values indicate better HRQL. Based on these domains, the physical component summary (PCS) and the mental component summary (MCS) can be calculated using norm-based scoring with a mean of 50. A score above 50 would be interpreted as better HRQL than the general Swedish population. The validation of the

For peer review only

Swedish SF-36 yielded Cronbach's α between 0.79 (role-emotional) and 0.93 (bodily pain) for the domains.[10]

The SF-36 profile in the study group was compared to a general population sample which was randomly selected from the Swedish SF-36 normative database (n=8,930; response rate 68%).[15] The normative sample (validated in Sweden 1991-92) was matched on sex and age and comprised 171 persons (141 males) with a mean age of 68.8 years (SD 11.1).

Patient and public involvement

Patients were not involved in the design, or conduct, or reporting, or dissemination plan of our research.

Results

Cohort characteristics and follow-up

The SF-36 questionnaire was returned by 223 ICD patients which corresponds to a response rate of 77.2%. The mean age was similar between those who responded and those who did not (p=0.276). The same was seen between sexes (p=0.255). Figure 1 depicts the flow-chart of the emerged cohort. The majority was men 82.1% (n=183) and 60.1% (n=134) had an ICD due to primary prevention. All leads in the device systems were implanted transvenously. The baseline characteristics of the patients are summarized in Table 1.

A total of 52 patients (23.3%) experienced appropriate ICD therapy, whereas 14 (6.3%) experienced inappropriate ICD shock during the follow-up period. Complications which required surgical intervention occurred in 39 patients (17.5%). Among patients with cardiomyopathy, 76 had ischemic disease and 56 had no known underlying ischemic disease.

For peer review only

The ages ranged from 32 to 90 years with a median age of 71.2 years. The mean age was 71.1 SD 9.7 years. Men and women had similar mean ages (71.1 vs 70.9 years). Similarly, the mean ages were not different for primary vs secondary prevention (70.3 vs 72.3 years; p=0.138), a history of appropriate ICD therapy (70.7 vs 72.3 years; p=0.286), inappropriate shock (71.1 vs 71.0 years; p=0.967), or a complication requiring surgery (71.1 vs 70.9 years; p=0.907). On the contrary, patients with ischemic cardiomyopathy were older than nonischemic cardiomyopathy patients (72.9 vs 66.9 years; p<0.001) as were patients with a history of AF at baseline (73.4 vs 70.1 years; p=0.024).

HRQL in the ICD cohort compared to general population norms

In six out of eight SF-36 domains, there was a significantly lower score in ICD patients compared to sex- and aged-matched population norms (Table 2 and Figure 2).

There was a statistically significant (p<0.05) difference for PF, RP, GH, VT, SF, and MH, but not for BP and RE. The effect size among the significantly lower domains ranged from 0.23 (RP) to 0.41 (GH). Both component scores were significantly lower and the effect sizes similar, PCS (0.31) and MCS (0.31). Thus, all effect sizes were classified as small. Men and women reported similar SF-36 scores with nonsignificant differences in all domains including the component scores; PCS 41.0 SD 12.4 vs 39.5 SD 12.0 (p=0.406) and MCS 48.7 SD 10.9 vs 48.3 SD 10.5 (p=0.644)

Increasing age was associated with significantly lower scores on PF (r=-0.36; p<0.001), RP (r=-0.25; p<0.001), BP (r=-0.20; p=0.003), RE (r=-0.16; p=0.017), and PCS (r=-0.28; p<0.001).

SF-36 scores for the following age strata: 32-59, 60-69, 70-79, and ≥80 years, are reported in Table 3. Kruskal-Wallis test showed significantly lower scores for PF, RP, and PCS, while BP and RE showed a trend toward lower scores.

For peer review only

HRQL subgroup analyses

The subgroup analyses for AF, complications, appropriate therapy, and inappropriate shocks are reported in Table 4. Patients with primary-prevention ICDs were compared to secondary prevention indications. The age was similar, 70.3 vs 72.3 years. Primary-prevention patients scored lower GH (p=0.016; ES 0.35) and there was a trend toward lower VT (p=0.097; ES=0.21), but otherwise no significant differences.

A history of AF at baseline was associated with significantly lower PF (p=0.004; ES=0.41) and a trend toward lower BP (p=0.083; ES=0.27) and VT (p=0.057; ES=0.31). This translated into lower PCS (p=0.013; ES 0.38) but similar MCS (p=0.928). However, the mean age of patients with AF was slightly higher (73.4 vs 70.1 years; p=0.024).

The mean age of patients who experienced appropriate ICD therapy patients was similar to patients who did not receive appropriate shocks (72.3 vs 70.7 years) and all SF-36 domains were similar without any significant differences or trends. The group who experienced inappropriate ICD therapy was small, but had a similar mean age and no significant differences or trends with regard to any SF-36 domain. Furthermore, the group who required surgical intervention due to a complication had a similar mean age and SF-36 scores without significant differences.

In the primary-prevention group, nonischemic vs ischemic cardiomyopathy were compared. Notably, patients with an ischemic etiology were older (72.9 vs 66.9 years) but no SF-36 domain showed significantly different scores (RP showed a trend of lower score in ischemic cardiomyopathy, p=0.067; ES=0.31).

DISCUSSION

For peer review only

This Swedish cohort of general ICD patients report worse HRQL than age- and sex-matched population norms. Except for BP and RE, all domains were significantly lower although effect sizes were considered small. The component score, PCS and MCS, showed an equally small effect size.

Similar component score

In a recently published Danish study based on unselected ICD recipients, PCS (41.1 SD 10.3) and MCS (45.7 SD 10.8) at a mean follow-up of 7 years, were similar to our findings; poor PF, RP, GH, and VT were associated with higher long-term mortality.[16] Early randomized controlled trials with shorter follow-up (1-3 years) reported similar findings.[17,18] In the MADIT II , PCS was associated with an 89% higher mortality, while MCS showed 39% higher mortality.[19] US data from the INTRINSIC RV trial found improvement of SF-score over the first year and at 12 months, PCS was 42.2 and MCS 51.1, which is also similar to our results.[20]

Heart failure and HRQL

Systolic heart failure was the ICD indication for the vast majority of patients in our cohort, a third of whom received a CRT-D device. Several studies have determined that heart failure is a main determinant of poor HRQL.[21]

In a systematic review reviewing many studies based on SF-36 poor physical health status predicted adverse coronary artery disease and HF prognosis.[21] Using both disease-specific and generic test instruments, HF studies clearly revealed poor HRQL, but poor mental status had little impact. Moreover, follow-up time did not influence the predictive value of health status. In another review, those who reported poor disease-specific health status, in particular HF patients, had increased mortality (hazard ratio 1.39) compared with those with moderate/good health status.[11] Systematic reviews in this field are hampered by severe

For peer review only - http://bmjopen.bmj.com/site/about/guidelines.xhtml

heterogeneity across populations, different follow-up times, and diverse outcome measurements. Still, HF has repeatedly been shown to be a major determinant of poor health status.[12,22] Underlying cardiac disease and comorbidities influence HRQL and mortality.[12,23] A Cochrane review of nonischemic HF concluded: "using ICD therapy probably has little or no effect on quality of life."[24] This view is further strengthened by the fact that various ICD patients without structural heart disease report similar SF-36 scores as the general populations.[25,26]

Atrial fibrillation

A systematic review of several studies demonstrated poorer HRQL in AF patients than healthy controls or general populations.[27] Notably, studies on AF and HRQL are diverse and often from specific cohorts as part of an evaluation of an intervention. Such studies are prone to selection bias because they likely reflect highly symptomatic patients. Nevertheless, more general AF patients likewise show lower HRQL.[28,29] With a qualitative exploratory approach, the various symptoms have been further elucidated.[30] AF patients consist of subgroups including those with paroxysmal, persistent, and permanent AF; and even within groups, the burden of symptoms may vary substantially. AF is common in patients with heart failure and thus prevalent in ICD cohorts. In our study, the comparison among those with/without a history of AF at baseline showed that the association of AF with poor HRQL was more pronounced for the physical domains, but this may be at least partly explained by older age.

Age

As expected, age correlated with lower HQRL scores on physical domains. The four age strata showed that PCS deteriorated with older age and demonstrated significantly lower

For peer review only

scores in the analyses. This is likely due to the burden of heart failure, AF, and other comorbidities. Interestingly, mental health domains were not affected and remained consistent throughout the age strata. It can be speculated that patients selected for ICD therapy reflect a group with relatively good mental health status.

Primary versus secondary indication

The proportion of primary prophylactic ICDs was low. This reflect a historical view on indication and possibly a more conservative approach towards primary prevention. Overall, the HRQL was similar and solely the GH domain was significantly lower in the primary prevention group. The coping strategy may differ between primary and secondary prevention patients. A survivor of a life-threatening arrhythmia may feel gratitude but be prone to anxiety and depression. A lower SF-36 score (all domains except BP) in primary vs secondary prevention has been shown, which underlines the vulnerability in the primary-prevention group.[31] The differences may also be attributed to baseline characteristics; HF is a decisive factor for ICD implantation and translates into worse HRQL scores. Secondary- vs primary- prevention patients are likely to have different expectations and acceptance of ICD therapy and its related complications.

ICD shocks and complications

In the three separate subgroup analyses with regard to appropriate ICD therapy, inappropriate ICD shock, and complications requiring surgery, no significant differences in the SF-36 domains were observed. This is somewhat reassuring, at least on a group level. Data from the INTRINSIC RV trial found no significant difference in HRQL between patients with and without ICD shocks.[20]

For peer review only

In a Danish cohort, HF was associated with poor scores on all SF-36 domains, while age and ICD shocks correlated less. Notably, on depression/anxiety-specific instruments, ICD shocks were a determinant for outcome. This suggests that it is the comorbidity burden, rather than the ICD itself, that determines the patients' HRQL and despite the fact that device-related complications can be distressing to individuals, ICDs are well tolerated overall. Of course, multiple ICD shocks may translate into worse HRQL, especially anxiety.[32] Our study was unable to detect a difference between patients with ICD shocks and those without. Several explanations have been suggested for this: lower incidence of shocks, ability to cope with complications over time, generic instruments not specifically designed for ICDs, and the overall security and reassurance that comes from an ICD. Thus, it should be highlighted that careful device programming and management to avoid shocks and complications are crucial for optimal care.

Gender perspectives

The vast majority (82%) of patients in our study were men. This is in line with a review of 19 studies, in which the percentage of men ranged from 54% to 83%. We found similar SF-scores in men and women. In another study, only PF and VT were lower among women, although the difference was small, while for women, NYHA-class and Type D personality were strong determinants of poor HRQL.[23] Women may be more prone to anxiety and to score lower also on some SF-36 domains (PF, SF, MH) regardless of a history of ICD shock.[33] There needs to be an increased awareness of female candidates for ICD therapy, and our study support the fact that there are no overall sex differences with regard to generic HRQL.

Clinical perspective

For peer review only

HRQL assessment has the potential to provide insights helpful to the overall management of ICD patients, including selection of ICD candidates, and the reduction of hospitalization and mortality.[34] Patients with ICDs have poorer general HRQL than the general population and increasing age and HF are strong determinants of worse HRQL. The underlying burden of comorbidities is the main driver for low HRQL rather than the ICD itself. Concerning quality of life improvement, overall management of the cardiac disease manifestations and comorbidities is important but challenging despite advances in many therapeutic fields. In fact, according to a meta-analysis there have been slight improvements regarding physical components, but not for mental components.[35] However, a comprehensive cardiac rehabilitation program in ICD patients improved both GH and MH.[34] Even though ICD shocks and complications can be devastating for an individual, the overall perception is that an ICD is generally well tolerated and does not affect generic HRQL. Individual assessment of benefits and risks, not gender category, should be part of the risk assessment strategy.

Furthermore, complications of ICD therapy do not have a significant impact on HRQL and should not be a reason to refrain from offering an ICD to otherwise eligible patients. Nevertheless, ICD is lifelong therapy and clinicians should regularly monitor the patient's ICD as well as quality of life as part of follow-up.

Limitations

The major strength of our study is the use of a "real-world" cohort, rather than strict inclusion/exclusion criteria used in randomized controlled trials, although this implies more heterogeneity than randomized controlled studies. The ICD patients included in our study come from a cohort of unselected patients without referral center bias. The SF-36 scores in the study group were compared with age- and sex-matched general population norms, that

For peer review only

although more than 20 years old, likely still represent current status. The observational design reflects common practice outside a clinical trial, but may hampered by lack of details in assessment of clinical profiles even though medical records were scrutinized. The proportion who responded to the survey was comparatively high, which implies less risk of differential bias.

The HRQL assessment comes from a cross-sectional cohort studied long after initial implant and likely reflects views from a more stabilized phase of life as an ICD patient. Nevertheless, the long-term impact on HRQL reported over the life of ICD patients remains unknown. We lack information about personality traits and socioeconomic factors, which may affect self-reported health. SF-36 is a well-validated questionnaire that allows for comparisons on a group level but is not specifically developed for disease-specific assessment. The study design has limitations with respect to determining causal pathways and merely show associations. Therefore, it should be interpreted with caution.

Conclusions

ICD patients have lower physical and mental HRQL, assessed by SF-36, than age- and sex-matched norms, although effect sizes are small. HRQL is similar regardless of sex, primary- or secondary-prevention indication, appropriate therapy, inappropriate shock, or complications but decreases with older age.

DECLARATIONS

Ethics: The study complies with the Declaration of Helsinki and has been approved by the Ethical Review Board in Uppsala (document number 2018/416).

Consent for publication: Not applicable.

For peer review only

Acknowledgements: The authors acknowledge editing by Jo Ann LeQuang of LeQ Medical who reviewed the manuscript for American English use. Thanks to Kristina Bergström for her help with data collection through the questionnaires. Region Gävleborg funded this research project.

Author contributions: Peter Magnusson: design, collection and interpretation of data, statistical analyses, writing the article. Gustav Mattsson: collection and interpretation of data, statistical analyses, critical revision writing the article. Marita Wallhagen: interpretation of data, critical revision. Jan Karlsson: statistical analyses, interpretation of data, critical revision. All authors approved the manuscript for submission.

Funding: This study had no external funding.

Competing interests: Peter Magnusson has received speaker fees or grants from Abbott, Alnylam, Amicus Therapeutics, Bayer, AstraZeneca, BMS, Boehringer-Ingelheim, Internetmedicin, Lilly, MSD, Novo Nordisk, Octopus Medical, Pfizer, Vifor Pharma, and Zoll.

Gustav Mattsson has received speaker fee from Alnylam, Internetmedicin, and MSD.

Marita Wallhagen and Jan Karlsson disclose no conflict of interest.

No external financial support or funding was received for this study.

Patient consent for publication: Not applicable.

Ethics approval: Approved by the Ethical Review Board in Uppsala (document number 2018/416)

Data sharing statement: All relevant data supporting the conclusions of this article is included within the article.

For peer review only

ORCID: Peter Magnusson: https://orcid.org/0000-0001-7906-7782

Gustav Mattsson: https://orcid.org/0000-0002-4317-0443

Marita Wallhagen: https://orcid.org/0000-0001-8413-3975

References

1. Priori SG, Blomström-Lundqvist C, Mazzanti A, et al. 2015 ESC Guidelines for the management of patients with ventricular arrhythmias and the prevention of sudden cardiac death: The Task Force for the Management of Patients with Ventricular Arrhythmias and the Prevention of Sudden Cardiac Death of the European Society of Cardiology (ESC). Endorsed by: Association for European Paediatric and Congenital Cardiology (AEPC). Eur Heart J 2015;36(41):2793–867.

2. Goldenberg I, Gillespie J, Moss AJ, et al. Long-term benefit of primary prevention with an implantable cardioverter-defibrillator: an extended 8-year follow-up study of the Multicenter Automatic Defibrillator Implantation Trial II. Circulation 2010;122(13):1265–71.

3. Smith T, Jordaens L, Theuns DAMJ, van Dessel PF, Wilde AA, Hunink MGM. The cost-effectiveness of primary prophylactic implantable defibrillator therapy in patients with ischaemic or non-ischaemic heart disease: a European analysis. Eur Heart J 2013;34(3):211–9.

4. Al-Khatib SM, Stevenson WG, Ackerman MJ, et al. 2017 AHA/ACC/HRS Guideline for Management of Patients With Ventricular Arrhythmias and the Prevention of Sudden Cardiac Death: A Report of the American College of Cardiology/American Heart Association Task Force on Clinical Practice Guidelines and the Heart Rhythm Society. J Am Coll Cardiol 2018;72(14):e91–220.

5. van der Heijden AC, Borleffs CJW, Buiten MS, et al. The clinical course of patients with implantable cardioverter-defibrillators: Extended experience on clinical outcome, device replacements, and device-related complications. Heart Rhythm 2015;12(6):1169–76.

6. Kirkfeldt RE, Johansen JB, Nohr EA, Jørgensen OD, Nielsen JC. Complications after cardiac implantable electronic device implantations: an analysis of a complete, nationwide cohort in Denmark. Eur Heart J 2014;35(18):1186–94.

7. Rector TS, Anand IS, Cohn JN. Relationships between clinical assessments and patients' perceptions of the effects of heart failure on their quality of life. J Card Fail 2006;12(2):87–92.

For peer review only

8. Luther SA, McCullough PA, Havranek EP, et al. The relationship between B-type natriuretic peptide and health status in patients with heart failure. J Card Fail 2005;11(6):414–21.

9. McHorney CA, Ware JE, Raczek AE. The MOS 36-Item Short-Form Health Survey (SF-36): II. Psychometric and clinical tests of validity in measuring physical and mental health constructs. Med Care 1993;31(3):247–63.

10. McHorney CA, Ware JE, Lu JF, Sherbourne CD. The MOS 36-item Short-Form Health Survey (SF-36): III. Tests of data quality, scaling assumptions, and reliability across diverse patient groups. Med Care 1994;32(1):40–66.

11. Mastenbroek MH, Versteeg H, Zijlstra W, Meine M, Spertus JA, Pedersen SS. Disease-specific health status as a predictor of mortality in patients with heart failure: a systematic literature review and meta-analysis of prospective cohort studies. Eur J Heart Fail 2014;16(4):384–93.

12. Hoogwegt MT, Kupper N, Jordaens L, Pedersen SS, Theuns DAMJ. Comorbidity burden is associated with poor psychological well-being and physical health status in patients with an implantable cardioverter-defibrillator. Europace 2013;15(10):1468–74.

13. Mattsson G, Magnusson P. Long-term follow-up of implantable cardioverter defibrillator patients with regard to appropriate therapy, complications, and mortality. Pacing Clin Electrophysiol 2020;43(2):245–53.

14. Cohen J. Statistical power analysis for the behavioral sciences. 2nd ed. Hillsdale, N.J: L. Erlbaum Associates; 1988.

15. Sullivan M, Karlsson J, Ware JE. The Swedish SF-36 Health Survey--I. Evaluation of data quality, scaling assumptions, reliability and construct validity across general populations in Sweden. Soc Sci Med 1995;41(10):1349–58.

16. van Veen B, Andersen CM, Johansen JB, Theuns DA, Pedersen SS. Patient-Reported Quality of Life as a Predictor of Mortality and Ventricular Tachyarrhythmia’s During 7 Years’ Follow-Up in Patients With an Implantable Cardioverter Defibrillator (from the MIDAS Study). Am J Cardiol 2019;123(4):605–10.

17. Steinberg JS, Joshi S, Schron EB, et al. Psychosocial status predicts mortality in patients with life-threatening ventricular arrhythmias. Heart Rhythm 2008;5(3):361–5.

18. Kao C-W, Friedmann E, Thomas SA. Quality of life predicts one-year survival in patients with implantable cardioverter defibrillators. Qual Life Res 2010;19(3):307–15.

19. Piotrowicz K, Noyes K, Lyness JM, et al. Physical functioning and mental well-being in association with health outcome in patients enrolled in the Multicenter Automatic Defibrillator Implantation Trial II. Eur Heart J 2007;28(5):601–7.

20. Gopinathannair R, Lerew DR, Cross NJ, Sears SF, Brown S, Olshansky B. Longitudinal changes in quality of life following ICD implant and the impact of age, gender, and ICD

For peer review only

shocks: observations from the INTRINSIC RV trial. J Interv Card Electrophysiol 2017;48(3):291–8.

21. Mommersteeg PMC, Denollet J, Spertus JA, Pedersen SS. Health status as a risk factor in cardiovascular disease: a systematic review of current evidence. Am Heart J 2009;157(2):208–18.

22. Johansen JB, Pedersen SS, Spindler H, Andersen K, Nielsen JC, Mortensen PT. Symptomatic heart failure is the most important clinical correlate of impaired quality of life, anxiety, and depression in implantable cardioverter-defibrillator patients: a single-centre, cross-sectional study in 610 patients. Europace 2008;10(5):545–51.

23. Habibović M, van den Broek KC, Theuns DAMJ, et al. Gender disparities in anxiety and quality of life in patients with an implantable cardioverter-defibrillator. Europace 2011;13(12):1723–30.

24. El Moheb M, Nicolas J, Khamis AM, Iskandarani G, Akl EA, Refaat M. Implantable cardiac defibrillators for people with non-ischaemic cardiomyopathy. Cochrane Database Syst Rev 2018;12:CD012738.

25. Probst V, Plassard-Kerdoncuf D, Mansourati J, et al. The psychological impact of implantable cardioverter defibrillator implantation on Brugada syndrome patients. Europace 2011;13(7):1034–9.

26. Christiaans I, van Langen IM, Birnie E, Bonsel GJ, Wilde AAM, Smets EMA. Quality of life and psychological distress in hypertrophic cardiomyopathy mutation carriers: a cross-sectional cohort study. Am J Med Genet A 2009;149A(4):602–12.

27. Thrall G, Lane D, Carroll D, Lip GYH. Quality of life in patients with atrial fibrillation: a systematic review. Am J Med 2006;119(5):448.e1-19.

28. Dorian P, Jung W, Newman D, et al. The impairment of health-related quality of life in patients with intermittent atrial fibrillation: implications for the assessment of investigational therapy. J Am Coll Cardiol 2000;36(4):1303–9.

29. van den Berg MP, Hassink RJ, Tuinenburg AE, et al. Quality of life in patients with paroxysmal atrial fibrillation and its predictors: importance of the autonomic nervous system. Eur Heart J 2001;22(3):247–53.

30. Stridsman M, Strömberg A, Hendriks J, Walfridsson U. Patients' Experiences of Living with Atrial Fibrillation: A Mixed Methods Study. Cardiol Res Pract 2019;2019:6590358.

31. Kikkenborg Berg S, Caspar Thygesen L, Hastrup Svendsen J, Vinggaard Christensen A, Zwisler A-D. Anxiety predicts mortality in ICD patients: results from the cross-sectional national CopenHeartICD survey with register follow-up. Pacing Clin Electrophysiol 2014;37(12):1641–50.

32. Redhead AP, Turkington D, Rao S, Tynan MM, Bourke JP. Psychopathology in postinfarction patients implanted with cardioverter-defibrillators for secondary

For peer review only

prevention. A cross-sectional, case-controlled study. J Psychosom Res 2010;69(6):555–63.

33. Spindler H, Johansen JB, Andersen K, Mortensen P, Pedersen SS. Gender differences in anxiety and concerns about the cardioverter defibrillator. Pacing Clin Electrophysiol 2009;32(5):614–21.

34. Kosiborod M, Soto GE, Jones PG, et al. Identifying heart failure patients at high risk for near-term cardiovascular events with serial health status assessments. Circulation 2007;115(15):1975–81.

35. Kao C-W, Chen M-Y, Chen T-Y, Lin P-H. Effect of psycho-educational interventions on quality of life in patients with implantable cardioverter defibrillators: a meta-analysis of randomized controlled trials. Health Qual Life Outcomes 2016;14(1):138.

For peer review only

● **Table 1.** Characteristics at ICD implant of 223 patients who completed the SF-36.

Patients	223
Mean age at implant	64.1 ± 10.9 years
Mean age at SF-36	71.1 ± 9.7 years
Females	40 (17.9%)
Device type	
ICD-VR	36 (16.1%)
ICD-DR	112 (50.2%)
CRT-D	75 (33.6%)
Hypertension	104 (46.6%)
Diabetes mellitus	47 (21.1%)
Renal failure*	27 (12.1%)
Atrial fibrillation	62 (27.8%)
Beta-blockers	201 (90.1%)
ACE-i/ARB	193 (86.5%)
MRA	104 (46.6%)

Data presented as frequencies (percentage in parenthesis).

ACE-I, angiotensin converting enzyme inhibitor; ARB, angiotensin receptor blockers; CRT-D, cardiac resynchronization therapy defibrillator; ICD-DR, dual lead implantable cardioverter defibrillator; ICD-VR, single lead implantable cardioverter defibrillator; MRA, mineralcorticoid receptor antagonist

*defined as S-Creatinine ≥130 µmol/L

For peer review only

• **Table 2:** SF-36 score in patients with ICDs compared to general Swedish population norms.

SF-36 domains	Cohort mean	SD	95% CI	Effect size	Norm mean	SD	95% CI	*p*-value
Physical functioning	65.1	28.1	61.4-68.8	0.38	75.5	26.0	71.5-79.6	**0.0002**
Role physical	56.9	44.1	50.9-62.8	0.23	66.7	40.4	60.2-73.2	**0.0466**
Bodily pain	71.9	29.9	67.9-75.8		71.0	27.2	66.9-75.1	0.6395
General health	56.2	21.7	53.3-59.1	0.41	65.9	25.3	61.9-70.0	**<0.0001**
Vitality	58.0	24.8	54.7-61.3	0.39	68.1	27.2	63.7-72.4	**<0.0001**
Social functioning	80.1	24.4	76.8-83.3	0.25	85.9	22.9	82.4-89.4	**0.0025**
Role emotional	72.2	39.9	66.9-77.6		76.7	35.7	71.1-82.4	0.3730
Mental health	76.2	19.4	73.6-78.8	0.38	83.8	20.9	80.5-87.2	**<0.0001**
Physical Component Summary	40.7	12.3	39.0-42.4	0.31	44.4	11.6	42.4-46.4	**0.0052**
Mental Component Summary	48.6	10.8	47.2-50.1	0.31	51.9	10.5	50.1-53.8	**0.0010**

For peer review only

● **Table 3:** SF-36 score in patients with implantable cardioverter defibrillators stratified by age.

	**32-59** (n=25)	**60-69** (n=72)	**70-79** (n=76)	**80+** (n=50)	
**SF-36 domains**	Mean (SD)	Mean (SD)	Mean (SD)	Mean (SD)	*p*-value*
**PF**	79.4 (25.4)	71.9 (26.3)	62.7 (29.0)	51.4 (24.4)	**<0.0001**
**RP**	80.0 (36.8)	60.0 (44.9)	56.0 (43.9)	40.9 (41.6)	**0.0050**
**BP**	80.8 (29.3)	74.4 (30.9)	71.5 (28.8)	64.2 (29.2)	0.0780
**GH**	57.5 (21.2)	56.0 (22.6)	56.2 (21.9)	55.7 (20.7)	0.9827
**VT**	64.6 (20.4)	59.7 (27.5)	56.5 (23.8)	54.3 (23.9)	0.2363
**SF**	84.0 (22.7)	79.3 (27.9)	80.3 (22.1)	78.8 (23.5)	0.8341
**RE**	82.7 (33.5)	75.6 (38.6)	73.6 (39.1)	59.7 (44.0)	0.0773
**MH**	77.8 (18.2)	77.2 (21.2)	74.4 (18.8)	76.8 (18.3)	0.6190
**PCS**	46.6 (11.1)	42.2 (12.7)	40.0 (11.9)	36.0 (11.4)	**0.0021**
**MCS**	48.6 (10.3)	48.3 (12.0)	48.6 (10.4)	49.3 (10.1)	0.9859

For peer review only

● **Table 4:** Subgroup analyses of ICD patients.

ES=effect size *Effect sizes were *lower* when atrial fibrillation was present.

**SF-36 domains**	**Atrial fibrillation***		**Complications**		**Appropriate therapy**		**Inappropriate shock**	
	p-value	ES	*p*-value	ES	*p*-value	ES	*p*-value	ES
Physical functioning	**0.004***	0.41	0.500		0.947		0.592	
Role physical	0.128		0.210		0.897		0.428	
Bodily pain	**0.083***	0.27	0.167		0.544		0.381	
General health	0.606		0.190		0.863		0.921	
Vitality	**0.057***	0.31	0.321		0.919		0.765	
Social functioning	0.669		0.910		0.943		0.944	
Role emotional	0.313		0.972		0.796		0.955	
Mental health	0.912		0.769		0.747		0.972	
Physical Component Summary	**0.013***	0.38	0.107		0.696		0.464	
Mental Component Summary	0.923		0.902		0.772		0.375	

For peer review only

Caption:

Figure 1. Flowchart.

Figure 2. Bar chart: Health-related quality of life among ICD patients compared to age- and sex-matched norms.

For peer review only

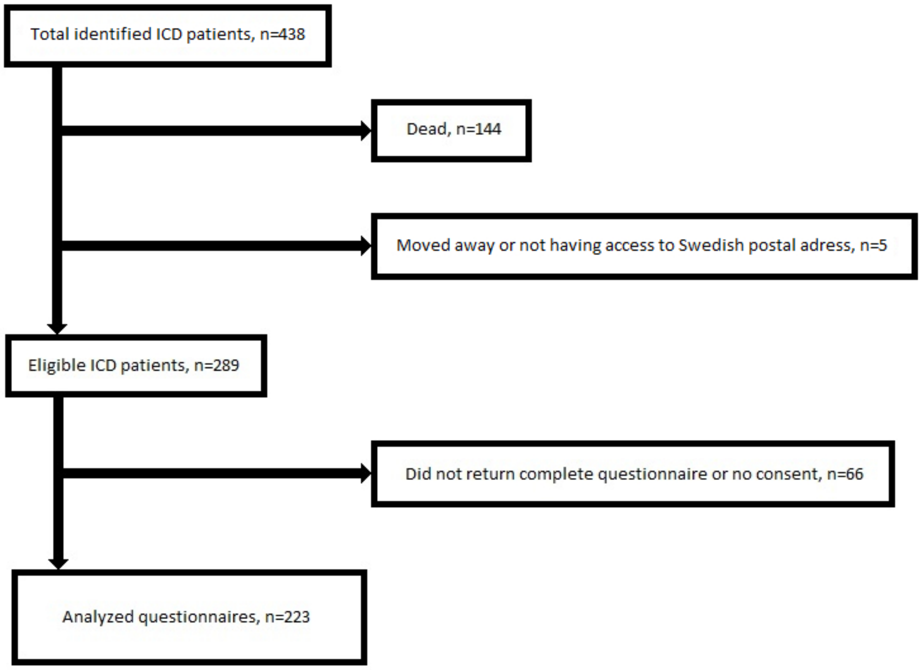

66x49mm (300 x 300 DPI)

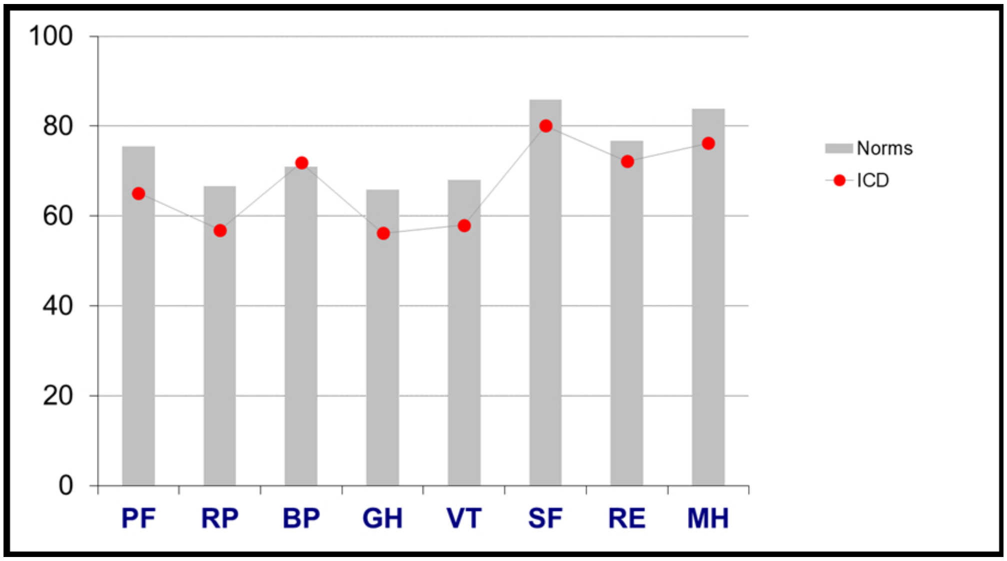
100
80
60
40
20
0
PF
RP
BP
GH
VT
SF
RE
MH
Norms
ICD

STROBE Statement—checklist of items that should be included in reports of observational studies

	Item No	Recommendation
**Title and abstract**	1	(*a*) Indicate the study's design with a commonly used term in the title or the abstract<br>Page 1
		(*b*) Provide in the abstract an informative and balanced summary of what was done and what was found<br>Page 2
**Introduction**		
Background/rationale	2	Explain the scientific background and rationale for the investigation being reported<br>Page 4-5
Objectives	3	State specific objectives, including any prespecified hypotheses<br>Page 4
**Methods**		
Study design	4	Present key elements of study design early in the paper<br>Page 5
Setting	5	Describe the setting, locations, and relevant dates, including periods of recruitment, exposure, follow-up, and data collection<br>Page 5
Participants	6	(*a*) *Cohort study*—Give the eligibility criteria, and the sources and methods of selection of participants. Describe methods of follow-up<br>Page 5<br>*Case-control study*—Give the eligibility criteria, and the sources and methods of case ascertainment and control selection. Give the rationale for the choice of cases and controls<br>*Cross-sectional study*—Give the eligibility criteria, and the sources and methods of selection of participants<br>Page 5
		(*b*) *Cohort study*—For matched studies, give matching criteria and number of exposed and unexposed<br>*Case-control study*—For matched studies, give matching criteria and the number of controls per case
Variables	7	Clearly define all outcomes, exposures, predictors, potential confounders, and effect modifiers. Give diagnostic criteria, if applicable<br>Page 5-6
Data sources/ measurement	8*	For each variable of interest, give sources of data and details of methods of assessment (measurement). Describe comparability of assessment methods if there is more than one group<br>Page 6
Bias	9	Describe any efforts to address potential sources of bias<br>Not applicable
Study size	10	Explain how the study size was arrived at<br>Not applicable
Quantitative variables	11	Explain how quantitative variables were handled in the analyses. If applicable, describe which groupings were chosen and why<br>Page 6

For peer review only

Statistical methods	12	(*a*) Describe all statistical methods, including those used to control for confounding<br>Page 6
		(*b*) Describe any methods used to examine subgroups and interactions<br>Page 6
		(*c*) Explain how missing data were addressed<br>Page 6
		(*d*) *Cohort study*—If applicable, explain how loss to follow-up was addressed<br>*Case-control study*—If applicable, explain how matching of cases and controls was addressed<br>*Cross-sectional study*—If applicable, describe analytical methods taking account of sampling strategy
		(*e*) Describe any sensitivity analyses

Continued on next page

For peer review only

**Results**		
Participants	13*	(a) Report numbers of individuals at each stage of study—eg numbers potentially eligible, examined for eligibility, confirmed eligible, included in the study, completing follow-up, and analysed<br>Page 8-9
		(b) Give reasons for non-participation at each stage<br>Yes, Figure 1
		(c) Consider use of a flow diagram<br>Yes, Figure 1
Descriptive data	14*	(a) Give characteristics of study participants (eg demographic, clinical, social) and information on exposures and potential confounders<br>Page 8
		(b) Indicate number of participants with missing data for each variable of interest<br>Figure 1
		(c) *Cohort study*—Summarise follow-up time (eg, average and total amount)
Outcome data	15*	*Cohort study*—Report numbers of outcome events or summary measures over time
		Case-control study—Report numbers in each exposure category, or summary measures of exposure
		Cross-sectional study—Report numbers of outcome events or summary measures<br>Page 8-9
Main results	16	*(a)* Give unadjusted estimates and, if applicable, confounder-adjusted estimates and their precision (eg, 95% confidence interval). Make clear which confounders were adjusted for and why they were included<br>*(b)* Figure 8-9
		(*b*) Report category boundaries when continuous variables were categorized
		(*c*) If relevant, consider translating estimates of relative risk into absolute risk for a meaningful time period
Other analyses	17	Report other analyses done—eg analyses of subgroups and interactions, and sensitivity analyses
**Discussion**		
Key results	18	Summarise key results with reference to study objectives<br>Page 11-15
Limitations	19	Discuss limitations of the study, taking into account sources of potential bias or imprecision. Discuss both direction and magnitude of any potential bias<br>Page 15
Interpretation	20	Give a cautious overall interpretation of results considering objectives, limitations, multiplicity of analyses, results from similar studies, and other relevant evidence<br>Page 16
Generalisability	21	Discuss the generalisability (external validity) of the study results<br>Page 16
**Other information**		
Funding	22	Give the source of funding and the role of the funders for the present study and, if applicable, for the original study on which the present article is based<br>Page 17

For peer review only

*Give information separately for cases and controls in case-control studies and, if applicable, for exposed and unexposed groups in cohort and cross-sectional studies.

Note: An Explanation and Elaboration article discusses each checklist item and gives methodological background and published examples of transparent reporting. The STROBE checklist is best used in conjunction with this article (freely available on the Web sites of PLoS Medicine at http://www.plosmedicine.org/, Annals of Internal Medicine at http://www.annals.org/, and Epidemiology at http://www.epidem.com/). Information on the STROBE Initiative is available at www.strobe-statement.org.

For peer review only

For peer review only - http://bmjopen.bmj.com/site/about/guidelines.xhtml
